# Variation in Suspected Cercospora Leaf Spot Incidence and Host Physiological and Fruit Traits Across Olive Tree Age Classes and Governorates in Palestinian Orchards

**DOI:** 10.3390/jof12100792

**Published:** 2026-10-21

**Authors:** Roba Al-Nazer, Besma Sghaier-Hammami

**Affiliations:** Laboratory of Bioaggressors and Integrated Pest Management in Agriculture (LR14AGR02), National Agronomic Institute of Tunisia, University of Carthage, 43 Avenue Charles Nicolle, Tunis 1082, Tunisia; roba.alnazer@gmail.com

**Keywords:** *Pseudocercospora cladosporioides*, *Olea europaea*, leaf spot disease, tree age class, relative water content, photosynthetic pigments, phenolic compounds, fruit characteristics, Palestine

## Abstract

Cercospora leaf spot, caused by *Pseudocercospora cladosporioides*, is a defoliating disease of olive. Symptomatic leaf-spot incidence and physiological, biochemical, and fruit traits were assessed in nine rain-fed orchards located in Bethlehem, Hebron, and Ramallah, Palestine. The surveyed trees belonged to the grower-identified cultivar ‘Roumi’ and were assigned to three broad age ranges (Range I, 50–100 years; Range II, 25–50 years; Range III, 15–20 years), resulting in six governorate × age-range groups. Symptomatic leaves were collected from all surveyed orchards, and fungal isolates were obtained and characterized by macroscopic and microscopic observations. The observed morphological characteristics were consistent with *Pseudocercospora cladosporioides*, although molecular confirmation was not performed. Mean symptomatic leaf-spot incidence ranged from 9.29% in Hebron Range I to 14.11% in Bethlehem Range I, with overlapping distributions and no monotonic pattern across age ranges. Within the shared 25–50-year age range, mean incidence was similar among governorates (9.95–11.02%), whereas RWC, total chlorophyll, and fruit weight showed differences among governorates. Exploratory orchard- and group-level correlations showed no clear association between symptomatic incidence and measured host traits. The first two principal components accounted for 67.9% of the variation among the six aggregated group means (PC1 = 44.0%; PC2 = 23.9%), with symptomatic incidence showing a near-zero loading on PC1 (−0.05), providing no clear evidence of an association with the principal physiological gradient. These observations provide a descriptive baseline for monitoring suspected Cercospora leaf spot and support future balanced, age-resolved surveys with molecular pathogen confirmation.

## 1. Introduction

Olive (*Olea europaea* L.) is one of the oldest cultivated fruit tree species and represents a cornerstone of agriculture, economy, and cultural heritage throughout the Mediterranean Basin. The region accounts for more than 90% of global olive production, where olive cultivation supports rural livelihoods, preserves traditional agroecosystems, and supplies high-quality olive oil and table olives. However, olive production is increasingly challenged by climate variability and numerous biotic constraints that threaten tree health, fruit quality, and orchard productivity [1,2,3]. In Palestine specifically, olive production and processing underpin rural livelihoods and remain a core component of the national agricultural economy [4,5]. Traditional Palestinian orchards contain trees ranging from recently established plantings to long-lived specimens that have remained productive for many decades, so regional field surveys frequently compare orchards that differ simultaneously in location, management, canopy architecture and age [6].

Among the fungal diseases affecting olive foliage, Cercospora leaf spot—also termed cercosporiosis or repilo plomizo—is a serious defoliating disease caused by *Pseudocercospora cladosporioides* (Sacc.) U. Braun when the causal agent is confirmed [7,8]. It must be clearly distinguished from peacock spot (olive leaf scab), the other major leaf-spotting disease of olive, which is caused by *Venturia oleaginea* (syn. *Spilocaea oleagina*, *Fusicladium oleagineum*) and frequently occurs in the same orchards [9]. Characteristic symptoms of confirmed cercosporiosis include light-green to yellow blotches on the upper leaf surface and leaden-grey or sooty sporulation on the lower surface [7,10]. Under favourable conditions, severe confirmed infections can lead to premature defoliation, reduced vegetative growth, lower flowering intensity, and yield losses [10,11]. Evidence from peacock spot, a comparable olive foliar disease caused by *V. oleaginea*, indicates that canopy conditions and inoculum retention in infected foliage influence disease development [12], whereas for cercosporiosis itself, moderate temperatures, prolonged leaf wetness and rainfall favour infection [8,13]. The disease has been reported across Mediterranean and temperate olive-growing regions, including Spain, Tunisia and the United Kingdom [14,15,16]; its incubation period may approach eleven months, and inoculum production is favoured by mild temperatures and high humidity [8,17]. The recent publication of a reference genome has strengthened the basis for molecular and epidemiological work on this pathosystem [18].

Despite substantial advances in understanding the epidemiology and management of olive foliar diseases, most studies have focused on pathogen biology, environmental conditions favouring infection, cultivar susceptibility, or disease control strategies [11,12]. Comparatively little attention has been devoted to the physiological responses of naturally symptomatic olive trees under field conditions, where disease expression occurs simultaneously with site, management and developmental influences. Consequently, it remains unclear how symptomatic leaf-spot incidence covaries with host physiological status across orchards differing in tree-age structure and location [1,2,19]. A further challenge is that governorate and tree age are often only partially crossed in existing orchard landscapes, creating a substantial risk of attributing age-structured variation to geography or vice versa [6]. A comparison within a shared age class can reduce one source of imbalance, but it cannot by itself estimate independent age and governorate effects.

Leaf water status, photosynthetic pigments, and phenolic compounds are widely recognized as indicators of plant physiological condition and stress responses. Leaf water content reflects plant water balance, chlorophyll and carotenoids determine photosynthetic efficiency and photoprotection, whereas phenolic compounds contribute to constitutive and inducible defence mechanisms against pathogens and abiotic stresses [20,21]. Pathogen infection commonly reduces chlorophyll concentration and photosynthetic capacity, although the magnitude varies with pathogen type and disease stage [22], and in olive nitrogen status, chlorophyll concentration, shoot growth and susceptibility to peacock spot can covary [23]. Phenolic compounds are abundant in olive tissues and contribute to defence-related metabolism [24]. Fruit characteristics integrate cumulative seasonal influences; fruit fresh weight is strongly heritable, but environmental conditions modify fruit expression, water status and phenolic composition [25,26,27], and photosynthate availability constrains fruit growth and ripening [28]. These traits have rarely been investigated simultaneously with suspected Cercospora-like leaf spot under natural orchard conditions. Studying one grower-identified cultivar reduces one source of genetic variation, but in this observational design differences in age, governorate and orchard conditions remain partially confounded and cannot be separated causally.

Palestine is one of the historical centers of olive cultivation, where olive production has considerable economic, ecological, and cultural importance. Earlier Palestinian surveys have quantified the incidence and severity of peacock spot across governorates and cultivars and reported associations with rainfall [29,30], but comparable field evidence for *P. cladosporioides*-associated leaf spot is largely absent. Comprehensive studies integrating symptomatic leaf-spot incidence with host physiological, biochemical, and pomological responses also remain scarce. Characterising these associations while explicitly accounting for the unbalanced age-by-governorate structure is important for designing more rigorous future monitoring and management studies in Mediterranean olive production systems.

Therefore, the objectives of this study were to (i) describe variation in suspected Cercospora leaf-spot incidence among the sampled governorate × age-range groups; (ii) explore relationships between symptomatic incidence and host physiological and biochemical traits, including leaf relative water content, photosynthetic pigments, total phenolic compounds, and fruit characteristics; and (iii) describe differences among governorates within a common broad age class (Range II). By integrating disease incidence, host functional traits and age-class structure under field conditions, this descriptive survey provided a field baseline on the occurrence of suspected Cercospora leaf spot in Palestinian olive orchards.

## 2. Materials and Methods

### 2.1. Study Area, Climate and Orchard Selection

The study was conducted in nine traditional rain-fed olive orchards in the central highlands of the West Bank, Palestine, distributed across Bethlehem, Hebron and Ramallah governorates, which differ in rainfall regime and atmospheric humidity. All orchards were managed under traditional rain-fed conditions with winter pruning and mechanical or manual weed control. No supplemental irrigation was applied at any site, and no foliar fungicide was applied during the eight weeks preceding the spring leaf-sampling campaign.

All sampled trees were identified by the growers as the local ‘Roumi’ cultivar, a traditional cultivar of the region; no molecular cultivar authentication was performed. Restricting the survey to one cultivar reduced one source of host genetic heterogeneity. Trees were classified into three operational age ranges on the basis of grower records and trunk architecture: Range I, approximately 50–100 years; Range II, approximately 25–50 years; and Range III, approximately 15–20 years. Bethlehem included Ranges I and II, Hebron included Ranges I, II and III, and Ramallah included Range II only, yielding six governorate × age-range groups: Bethlehem_RangeI, Bethlehem_RangeII, Hebron_RangeI, Hebron_RangeII, Hebron_RangeIII and Ramallah_RangeII. Exact individual-tree ages were not recorded in the analytical dataset, so within-class age distributions cannot be described or shown to be balanced among governorates. In particular, Range II spans approximately 25 years and should be interpreted as broad age-class matching rather than exact age standardisation; narrowing the class would require new age-resolved sampling.

Meteorological records for the three governorates were obtained from the national station network operated by the Palestinian Meteorological Directorate and compiled by the Palestinian Central Bureau of Statistics [31]. Monthly mean air temperature, mean relative humidity and total rainfall for January–September 2024, i.e., the growing season that preceded the October–November 2024 fruit harvest, are summarized in Table 1. These station-level records therefore describe the season in which the sampled fruit developed, but they do not cover the period between the harvest and the spring leaf campaign (October 2024–April 2025). They are used as governorate-level climatic context only and are not treated as orchard-level measurements or as explanatory covariates. Bethlehem temperature and relative-humidity values were reconstructed by interpolation between the flanking Ramallah and Hebron stations, whereas the Bethlehem rainfall series was obtained directly.

All three governorates share a Mediterranean climate of the Köppen Csa type, with a cool, wet winter and a hot, rain-free summer. In the 2024 record, rainfall was concentrated between January and March and was nil from June to August, while monthly mean temperature rose from approximately 13 °C in January–February to above 30 °C in June–August. Over January–September 2024, cumulative rainfall was 451.9 mm in Ramallah, 411.5 mm in Bethlehem and 306.2 mm in Hebron, while mean relative humidity was 66.0%, 69.1% and 72.2%, respectively [31]. Mean air temperature was approximately 23.1 °C in all three governorates. Longer-term reference mean annual rainfall is 615.2 mm for Ramallah and Al-Bireh, 595.9 mm for Hebron and 518.4 mm for Bethlehem [31]. These values describe governorate-level conditions during the 2024 fruit-growing season; because they are station records rather than orchard measurements and do not extend to the spring 2025 leaf campaign, they are not used to attribute the disease, physiological or fruit measurements to rainfall, humidity or temperature.

### 2.2. Disease Survey and Sampling Strategy

Two linked sampling campaigns were completed on the same tagged trees. Five representative trees of the target age range were tagged in each of the nine orchards, giving 45 tagged trees in total. Mature fruits were collected at commercial maturity (approximately maturity index 3–4) during 20–28 October 2024 in Bethlehem, 2–11 November 2024 in Hebron and 14–18 November 2024 in Ramallah. Spring leaf sampling was conducted on the same tagged trees during 18–26 March 2025 in Bethlehem, 30 March–13 April 2025 in Hebron and 19–28 April 2025 in Ramallah. Thus, leaf sampling was not simultaneous among governorates and spanned approximately six weeks from the earliest Bethlehem to the latest Ramallah collection. Because relative water content, pigments and phenolics may vary during spring development, sampling date is a potential governorate-level confound and is considered in interpretation and limitations.

Leaves and fruits were collected from all four canopy aspects at approximately 1.5–2.0 m above ground level. Samples were labelled by site and tree, protected from direct sunlight, transported in insulated containers and processed within six hours of collection. Sampling intensity was standardised for every tagged tree: ten branches distributed over the four canopy aspects were selected for assessment of suspected Cercospora leaf-spot symptoms; ten fully expanded leaves per tree were collected for leaf relative water content and photosynthetic pigments; 100 leaves per tree were collected for total phenolic content; and approximately 60–75 fruits per tree were collected for pomological assessment. Leaves intended for biochemical assays were pooled per tree before subsampling. Branches and fruits collected from the same tree are subsamples rather than independent experimental units, and trees are themselves clustered within orchards. Table 2 therefore reports measurement records/subsamples, not independent replicate counts.

### 2.3. Morphological Assessment and Isolation of Fungi Associated with Suspected Cercospora Leaf Spot

Symptomatic leaves showing upper-surface blotching and lower-surface leaden-grey sporulation were collected from every orchard. Sporulating tissue was gently scraped directly from the abaxial leaf surface into a drop of lactophenol cotton blue on a glass slide, covered with a coverslip and examined with an Olympus CX23 compound bright-field microscope (Olympus Corporation, Tokyo, Japan) fitted with a 10× eyepiece. Conidiophore fascicles and conidial masses were examined with the 10× objective (100× total magnification), and septation together with individual conidial morphology was examined with the 40× objective (400× total magnification). Observed structures included pigmented, septate conidiophores bearing hyaline to pale-olivaceous, elongate, multiseptate conidia, consistent with published descriptions of *P. cladosporioides* [7,8]. No ITS amplification, sequencing or other molecular confirmation was performed. Accordingly, the study reports a morphological association with *P. cladosporioides* rather than definitive species-level confirmation of the causal agent.

Fungal isolation was also performed from the same symptomatic leaves at the laboratories of Hebron University. Small leaf pieces (approximately 5 × 5 mm) were cut from the margins of sporulating lesions, surface-disinfected in 1% sodium hypochlorite for 1 min, rinsed three times in sterile distilled water, blotted dry on sterile filter paper and plated on potato dextrose agar (PDA) amended with streptomycin sulphate (0.5 g L^−1^).

Plates were incubated at 25 ± 1 °C in darkness. Hyphal tips from the margins of developing colonies were transferred to the center of fresh PDA plates to obtain pure cultures, which were incubated under the same conditions for 21 days. In total, 21 isolates obtained from symptomatic leaves collected in the three governorates were examined, and colony pigmentation, surface texture, aerial mycelium, radial sectoring and growth pattern were recorded. The cultured isolates were examined macroscopically only and were not linked microscopically to the structures observed on the leaves. The morphological assessment relied on the conidiophores and conidia observed directly on sporulating lesions.

A note on nomenclature is required because the vernacular term “olive leaf spot” has been applied in the literature to two distinct diseases. Molecularly or otherwise definitively confirmed *Pseudocercospora cladosporioides* (Mycosphaerellaceae, Capnodiales) causes Cercospora leaf spot of olive, whereas *Venturia oleaginea* (Venturiaceae, Venturiales; asexual morphs *Spilocaea oleagina* and *Fusicladium oleagineum*) causes peacock spot or olive leaf scab [7,8,9,14]. During the present survey, leaves bearing only adaxial concentric lesions typical of peacock spot were not scored as cases of the target symptomatic syndrome; scoring required the characteristic abaxial grey-sooty sporulation together with compatible conidial morphology. This operational distinction reduces, but cannot eliminate, diagnostic uncertainty. Because *V. oleaginea* can co-occur on the same tree or even on the same leaf and no lesion-level molecular testing was performed, mixed infection cannot be excluded. Study-specific disease labels are therefore qualified as “suspected Cercospora leaf spot” or “symptomatic leaf spot” throughout.

### 2.4. Assessment of Suspected Cercospora Leaf-Spot Incidence

Symptomatic leaf-spot incidence was assessed on ten branches per tree, selected to represent the four canopy aspects. All leaves borne on each branch were examined on both surfaces under diffuse light and classified as asymptomatic or symptomatic for the target syndrome; a leaf was scored as symptomatic when it displayed compatible yellow blotching, necrosis and grey-sooty abaxial sporulation. This field classification was an operational morphological diagnosis and should not be interpreted as molecular confirmation of *P. cladosporioides* infection. The symptomatic incidence was calculated for each branch as:

Symptomatic leaf-spot incidence (%) = (*N*_i_/*N*_t_) × 100 (1)

where *N*_i_ is the number of leaves showing the target leaf-spot symptoms and *N*_t_ is the total number of leaves assessed on the branch, that is the sum of asymptomatic and symptomatic leaves. Records that could not be scored reliably, for example because of storm damage or premature defoliation, were excluded, and 429 valid branch-level records were retained for descriptive summaries (Table 2). The variable quantified here is symptomatic leaf incidence, not diseased leaf area; incidence-based and severity-based indices are therefore not directly interchangeable.

### 2.5. Determination of Host Physiological and Biochemical Traits

#### 2.5.1. Leaf Relative Water Content

Leaf relative water content (RWC) was determined on the ten fully expanded leaves collected from each tagged tree, from which 58 samples were prepared. Fresh mass was recorded immediately using a calibrated analytical balance with 0.001 g readability. Leaves were then floated on distilled water in closed Petri dishes and held in darkness at 4 °C for 24 h to reach full turgor, blotted rapidly with absorbent paper and reweighed to obtain the turgid mass. Samples were dried in a forced-air oven at 70 °C for 48 h and reweighed to obtain the dry mass; samples that had not reached constant mass were returned to the oven for additional 6 h intervals until two consecutive measurements differed by less than 1%. Relative water content was calculated as:

RWC (%) = [(FW − DW)/(TW − DW)] × 100(2)

where FW is the fresh mass recorded immediately after collection (g), TW is the turgid mass reached after saturation (g) and DW is the dry mass obtained after oven drying to constant mass (g).

#### 2.5.2. Chlorophyll and Carotenoid Determination

Photosynthetic pigments were quantified in 38 fresh-leaf samples prepared from the ten leaves collected per tree, following the method of Lichtenthaler [32]. Fresh leaf tissue (0.5 g) was introduced into vials containing 10 mL of 80% (*v*/*v*) acetone; vials were covered with aluminium foil and kept in darkness at 4 °C for 72 h. Absorbance was then read at 470, 646 and 663 nm with a Dual Beam 8 Auto Cell UVS-2700 UV–visible spectrophotometer (Labomed, Inc., Los Angeles, CA, USA), using 80% acetone as the blank; one extract with an absorbance above the linear range was read after two-fold dilution. Pigment concentrations were calculated as:

Chl *a* (µg mL^−1^) = (12.21 × *A*_663_) − (2.81 × *A*_646_) (3)

Chl *b* (µg mL^−1^) = (20.13 × *A*_646_) − (5.03 × *A*_663_) (4)

Carotenoids (µg mL^−1^) = [(1000 × *A*_470_) − (3.27 × Chl *a*) − (104 × Chl *b*)]/227 (5)

Total Chl (µg mL^−1^) = (6.45 × *A*_663_) + (17.27 × *A*_646_) (6)

where *A*_470_, *A*_646_ and *A*_663_ are the absorbances read at the corresponding wavelengths. Total chlorophyll was calculated directly from Equation (6) and not as the sum of chlorophyll *a* and *b* from Equations (3) and (4); consequently, the sum of the chlorophyll *a* and *b* fractions (Table S3) is approximately 5–9% higher than the total chlorophyll reported here. All pigment concentrations were converted from µg mL^−1^ to µg g^−1^ fresh weight by multiplying by the extraction volume (10 mL) and the dilution factor and dividing by the fresh mass (0.5 g). All values reported in this article were recalculated from the original absorbance readings using Equations (3)–(6) without any subsequent rescaling. One Bethlehem Range I extract returned a negative chlorophyll *b* estimate and one Ramallah Range II extract a negative carotenoid estimate; because such values are physically impossible, they were excluded from the chlorophyll *b*, carotenoid, chlorophyll *a*/*b* and carotenoid:chlorophyll summaries (*n* = 37 for chlorophyll *b* and *n* = 36 for carotenoids), whereas total chlorophyll, which does not depend on these terms, was retained for all 38 extracts.

#### 2.5.3. Total Phenolic Content

The 100 leaves collected per tree for biochemical determination were oven-dried and ground to a fine powder. Extraction followed the method adapted from Mau et al. [33]: 1 g of dry matter was weighed, 10 mL of pure methanol was added, and the mixture was agitated for 30 min before filtration through ash-free filter paper; the filtrate was stored at 4 °C. The available laboratory record does not establish whether an additional 24 h hold at 4 °C in darkness was applied uniformly across all extracts; accordingly, this interval is not specified as a standardized step in the protocol and the procedural uncertainty is considered in Section 4.6. Total phenolic content was quantified in 48 samples with the Folin–Ciocalteu colorimetric assay [34] as described by Dewanto et al. [35]. For each determination, 125 µL of suitably diluted extract, 500 µL of distilled water and 125 µL of Folin–Ciocalteu reagent were added to a test tube; the tube was shaken vigorously and left to stand for 3 min, after which 1250 µL of 7% (*w*/*v*) sodium carbonate solution and 1000 µL of distilled water were added. Tubes were covered with aluminium foil and incubated for 90 min at room temperature in darkness, and absorbance was read at 760 nm. A calibration curve was prepared with gallic acid at 50, 100, 200, 300 and 400 mg L−1, and results were expressed as milligrams of gallic acid equivalents per gram of dry matter (mg GAE g−1 DW).

### 2.6. Fruit Characterization

Fruit traits were measured on mature fruit collected during the October–November 2024 harvest from the same tagged trees used for the spring leaf campaign. After exclusion of damaged fruit, 2989 individual fruits were retained for morphometric assessment. Longitudinal length and maximum equatorial diameter were measured with a digital vernier calliper (0.01 mm resolution), and the fruit-size index was defined as length multiplied by equatorial diameter after conversion to centimetres, expressed in square centimetres. Fruit weight, stone-to-pulp ratio and pulp water content were determined from 56 ten-fruit composite subsamples. One composite was prepared from each sampled tree where possible, and additional composites were prepared from some trees when sufficient sound fruit was available; such additional composites remain subsamples from the same tree and are not independent biological replicates. Whole fruits were weighed on a balance with 0.001 g readability. Pulp was manually separated from the stone, the stone was blotted before weighing, pulp fresh mass was calculated as whole-fruit mass minus stone mass, and the stone-to-pulp ratio was calculated as 100 × stone fresh mass divided by pulp fresh mass. Pulp water content was determined by drying at 70 °C for 48 h to constant mass and calculated on a fresh-mass basis as 100 × (fresh mass − dry mass) divided by fresh mass.

### 2.7. Statistical Analysis

All analyses were performed in Python 3.11 (Python Software Foundation, Wilmington, DE, USA) using SciPy, statsmodels, pandas, NumPy and Matplotlib (version 3.10). Descriptive statistics comprised the number of records, mean, standard deviation and coefficient of variation in each governorate × age-range group (Table S1), and pairwise differences between group means are listed descriptively in Table S2. The sampling structure is hierarchical: branches and individual fruits are nested within trees, trees are nested within orchards, and each governorate × age-range group was represented by only one or a few of the nine orchards. Although samples were labelled by site and tree in the field, these identifiers were not retained for all records in the analytical dataset, so complete tree- and orchard-level linkage is unavailable for the 429 branch records and 2989 individual-fruit records; therefore, a valid hierarchical (e.g., mixed-effects) analysis could not be reconstructed. For this reason, the study is presented as a descriptive observational survey, and no inferential tests, effect sizes, post hoc comparisons or *p* values are used to compare governorate × age-range groups. Branches, individual fruits, leaf extracts and additional composite fruit subsamples from the same tree are not treated as independent biological replicates; differences among groups are described from group means, standard deviations and the overlap of record distributions.

Within this descriptive framework, a focused comparison was made within Range II, the broad 25–50-year age class represented in all three governorates. This is an age-class-restricted governorate comparison only; it is neither exact age standardization nor statistical control of age, and it cannot estimate independent age and governorate effects. Exploratory Spearman rank correlations were calculated only from aggregated values, so that branch, fruit and extract records were not treated as independent observations: orchard (site)-level means were used for variables available from matched orchards (*n* = 7–9 orchards, depending on the variable), and the six governorate × age-range group means (*n* = 6) were used when total chlorophyll and carotenoids were included and for the full correlation matrix. Given these very small sample sizes, correlation coefficients and their nominal *p* values are reported only as descriptive indications of direction and strength. Principal component analysis (PCA) was performed on the standardized matrix of six group means × nine variables solely to visualize these aggregated means; with six observations and nine variables, the ordination is unstable and is not used to infer biological independence or to support other conclusions. Associations linking fruit traits from the 2024 harvest with leaf traits from spring 2025 are cross-seasonal rather than simultaneous.

### 2.8. Generative AI Assistance in Manuscript Preparation

ChatGPT 5 (OpenAI, San Francisco, CA, USA) was used for English-language proofreading, journal-format organization and editorial refinement. Experimental observations, measurements and original study micrographs were not generated or altered. Scientific interpretation and responsibility for the final content remain with the authors.

## 3. Results

All surveyed orchards belonged to the same grower-identified olive cultivar (‘Roumi’), reducing one source of host heterogeneity but not eliminating differences in orchard management, soil, canopy structure, microclimate, sampling date or tree age. The dataset spans nine orchards (45 tagged trees) in Bethlehem, Hebron and Ramallah and six observed governorate × age-range groups: Bethlehem_RangeI, Bethlehem_RangeII, Hebron_RangeI, Hebron_RangeII, Hebron_RangeIII and Ramallah_RangeII. Ramallah is represented only in Range II, Bethlehem in Ranges I and II, and Hebron in all three ranges. Consequently, governorate and age are not factorially crossed, and differences among the six groups cannot be independently attributed to age or governorate. The analyses below describe group patterns and an age-class-restricted Range II comparison; for the reasons stated in Section 2.7, all group comparisons are descriptive, and no inferential tests are applied to the nested branch, fruit or extract records.

### 3.1. Morphological Assessment of Fungal Structures Associated with Suspected Cercospora Leaf Spot

**Figure 1:**
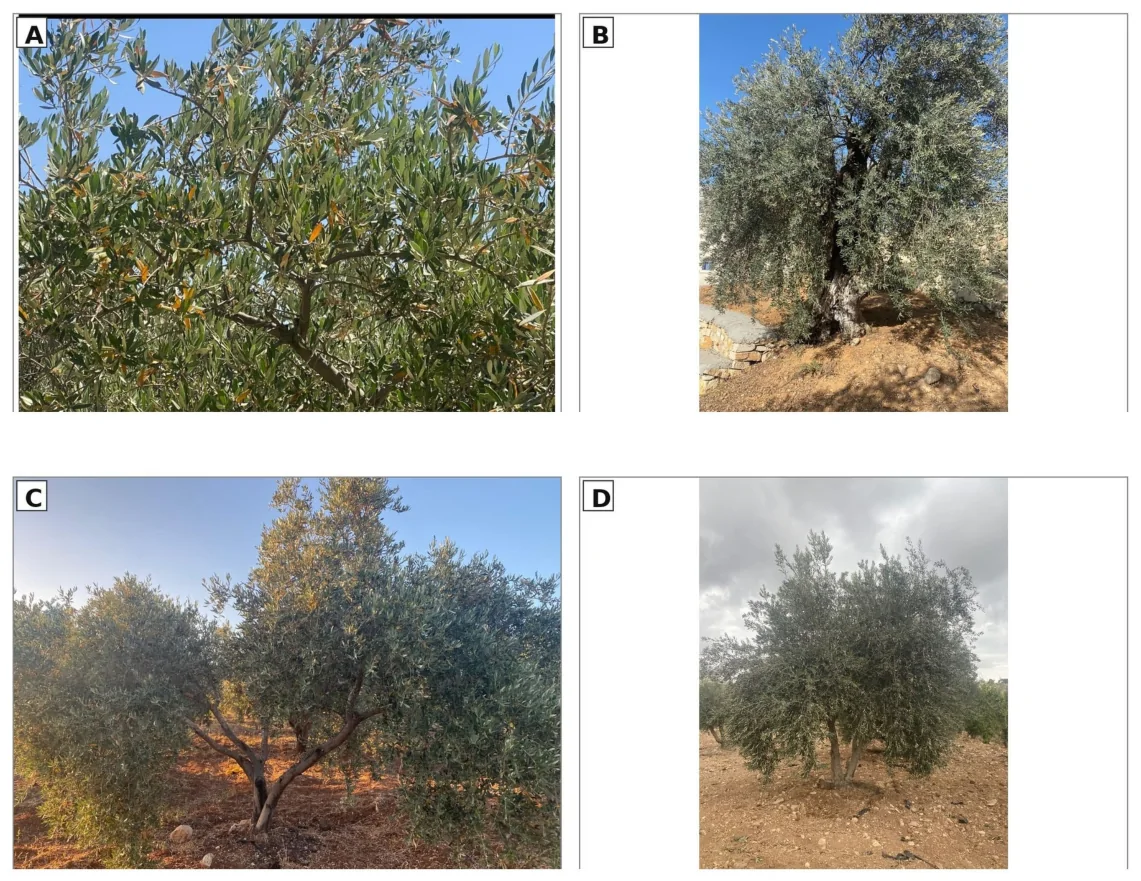
Representative rain-fed olive trees included in the field survey. Panels (**A**–**D**) illustrate variation in canopy architecture and orchard setting; locality labels are omitted because the photographs are illustrative rather than a direct regional comparison.

Before comparing symptomatic leaf-spot incidence among governorates and age classes, symptomatic leaves were examined to determine whether the associated fungal structures were morphologically compatible across orchards. The surveyed trees and their orchard settings are illustrated in Figure 1. Macroscopic observations showed a recurring syndrome of light-green to yellow adaxial blotching with olive-grey to sooty abaxial sporulation. These symptoms are compatible with cercosporiosis but are not, by themselves, species-specific.

**Figure 2:**
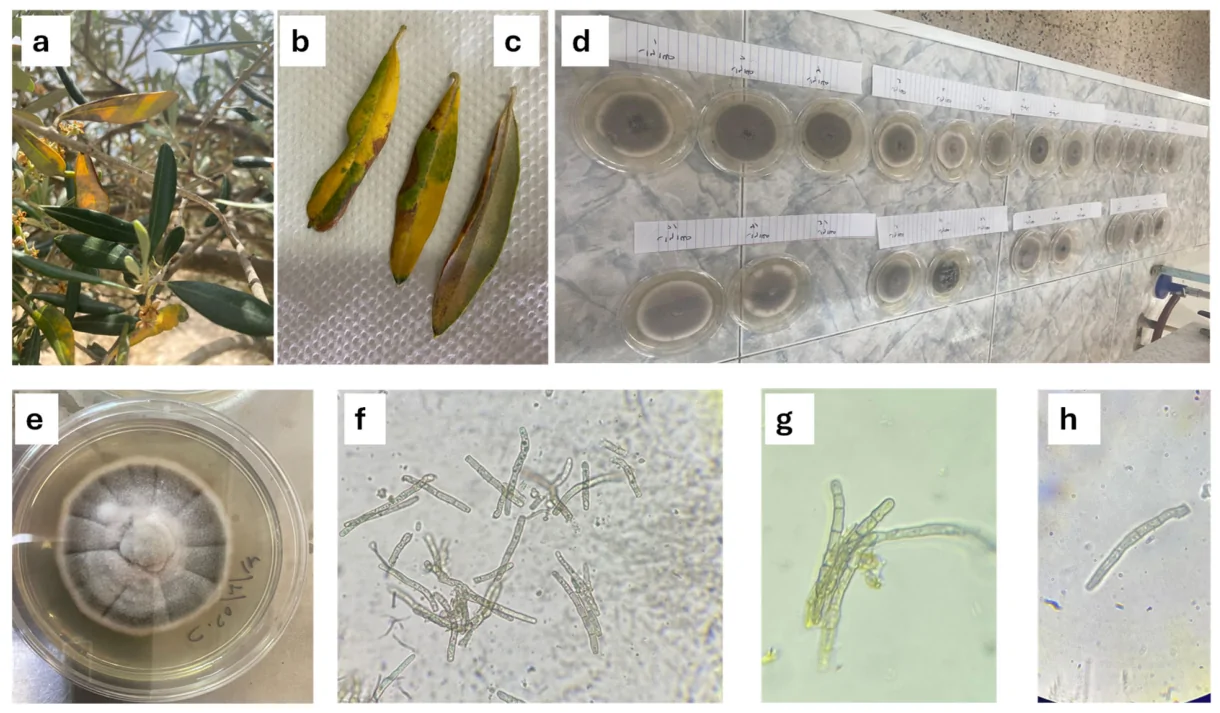
Field symptoms and morphological characteristics of fungi associated with suspected Cercospora leaf spot of olive. (**a**) Olive leaves showing leaf-spot symptoms directly on the tree. (**b**) Adaxial surface of symptomatic leaves collected from different governorates, showing chlorotic yellowing and brown necrotic lesions. (**c**) Abaxial surface of a symptomatic leaf showing greyish to dark sporulation. (**d**) Fungal isolates obtained on PDA from symptomatic leaves collected from different governorates, showing broadly similar colony morphology with variation in colour, texture, aerial mycelium, and growth pattern. (**e**) Representative fungal colony showing dark olive-grey pigmentation, radial sectoring, and a whitish aerial mycelial margin. (**f**–**h**) Direct microscopic observations of sporulating lesions showing septate, pigmented conidiophores and elongated, hyaline to pale-olivaceous, multiseptate conidia, ranging from straight to slightly curved. The microscopic characteristics (**f**–**h**) were morphologically consistent with *Pseudocercospora cladosporioides*; the culture images (**d**,**e**) document fungal growth from symptomatic tissue and are not by themselves diagnostic. No molecular identification was performed. The handwritten labels on the plates in (**d**) are in Arabic and record the locality of origin and the isolate number of each plate, and the handwritten marks on the plate in (**e**) are laboratory plate annotations; these labels are not required for interpretation of the figure.

Leaves observed directly on the trees showed characteristic leaf-spot symptoms, including chlorotic to yellowish discoloration and brown to dark necrotic lesions (Figure 2a). Symptomatic leaves collected from different governorates exhibited similar symptoms, with brownish to dark lesions and greyish to dark sporulation, particularly on the abaxial surface (Figure 2b,c). Isolation from these symptomatic leaves yielded cultures showing broadly similar macroscopic characteristics, although some variation in colony colour, texture, aerial mycelium, and growth pattern was observed among isolates (Figure 2d). A representative isolate showed a dark olive-grey colony with radial sectoring, a whitish aerial mycelial margin, and a compact central growth zone (Figure 2e). Direct microscopic examination of sporulating lesions revealed septate, pigmented conidiophores and elongated, hyaline to pale-olivaceous, multiseptate conidia, which were straight to slightly curved and variable in shape (Figure 2f–h). These morphological characteristics were consistent with published descriptions of *Pseudocercospora cladosporioides* [7,8]. Because cultural characteristics are not species-diagnostic and neither the isolates nor the lesions were molecularly identified, the culture images are presented as supporting documentation only; the morphological compatibility with *P. cladosporioides* rests on the structures observed directly on symptomatic leaves.

### 3.2. Variation in Suspected Cercospora Leaf-Spot Incidence Among Governorates and Tree Age Classes

Descriptive statistics for the nine analytical domains are presented in Table 3, with the numbers of records and coefficients of variation in Table S1. In accordance with Section 2.7, differences among the six governorate × age-range groups are described from group means, standard deviations and the distributions of records, without inferential tests.

**Figure 3:**
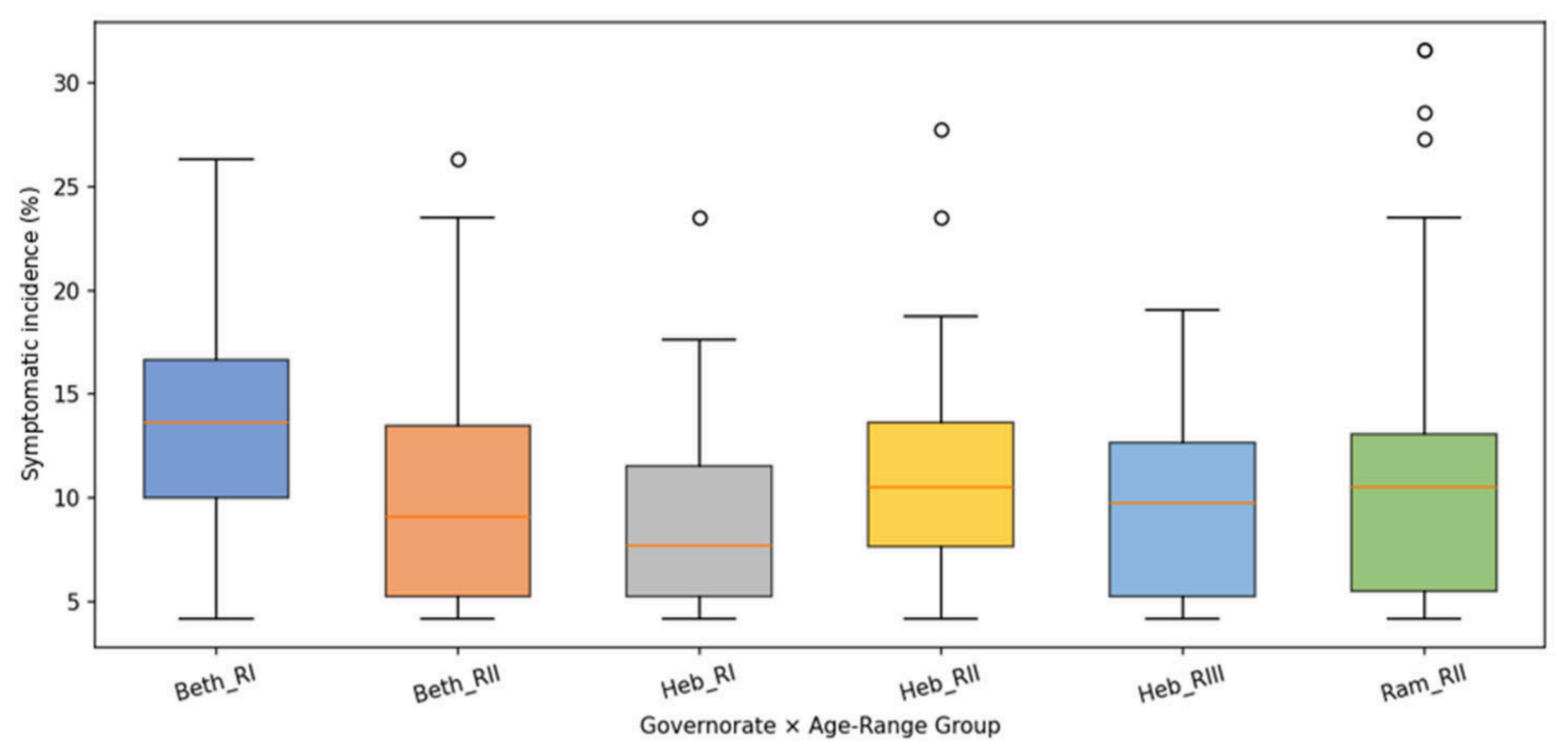
Boxplot of symptomatic leaf-spot incidence (%) by governorate × age-range group (429 branch-level records). Boxes show the interquartile range and median, whiskers the 1.5 × IQR limits, and circles outlying branch records. Because branches are nested within trees and orchards, the boxes summarize subsamples rather than independent biological replicates; no inferential test is shown.

Following the morphological assessment, symptomatic leaf-spot incidence was summarized across the six governorate × age-range groups using 429 branch-level records (Table 3; Figure 3). Mean symptomatic incidence ranged from 9.29% in Hebron Range I to 14.11% in Bethlehem Range I; the remaining four groups had means between 9.93% and 11.02%. Bethlehem Range I also had the highest median, but the branch-record distributions of all six groups overlapped widely, and the within-group standard deviations (4.11–5.73 percentage points) were comparable to or larger than the differences between group means. Because branches are nested within trees and trees within orchards, these records are not independent replicates and are summarized descriptively. The group pattern was also non-monotonic with age: Hebron Range II had a higher mean than Hebron Ranges I and III, whereas Bethlehem Range I exceeded Bethlehem Range II. Accordingly, the six-group comparison does not indicate a general tree-age pattern.

Because age classes were unevenly represented among governorates, symptomatic incidence was also described among governorates within Range II, the only broad age class represented in all three locations. Mean incidence was 9.95 ± 5.07% in Bethlehem, 10.89 ± 4.67% in Hebron and 11.02 ± 5.70% in Ramallah (Table 3 and Table S4); the three means differed by about one percentage point, and the branch-record distributions overlapped extensively (Figure 3). Thus, the higher mean incidence in Bethlehem Range I was not paralleled by a governorate difference within this shared broad age class. Because exact ages were heterogeneous within Range II, age and governorate were not independently crossed, and each group comprised few orchards, the design cannot determine why the six-group and Range II comparisons differed.

To examine whether host-trait patterns paralleled symptomatic leaf-spot incidence, the following sections describe leaf water status, photosynthetic pigments, phenolic compounds and fruit traits across the observed governorate × age-range groups, with the same restrictions on causal and inferential interpretation noted above.

### 3.3. Variation in Host Water Status Among Governorates and Tree Age Classes in Relation to Symptomatic Leaf-Spot Incidence

**Figure 4:**
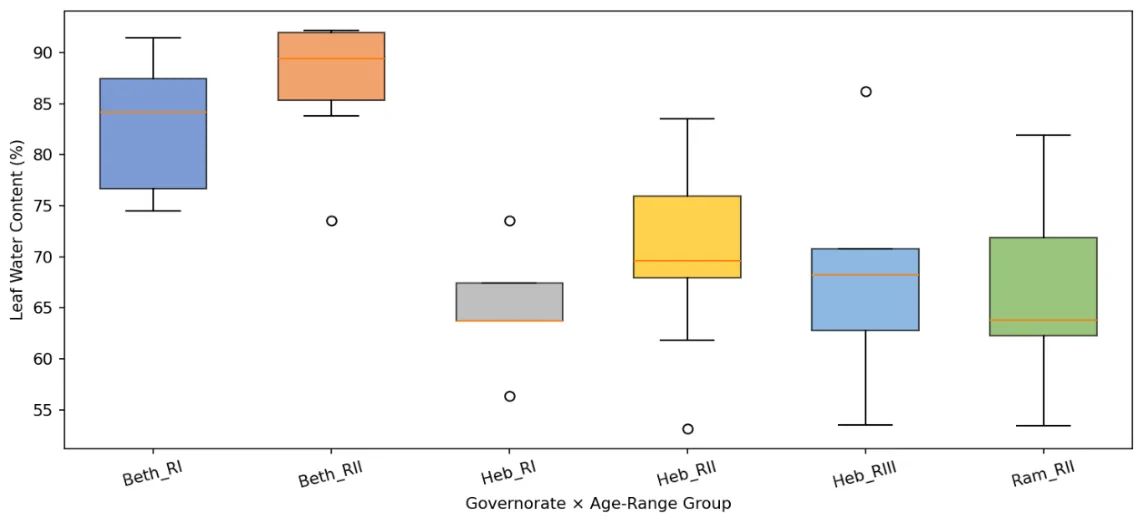
Boxplot of leaf relative water content (%) by governorate × age-range group. Boxes show the interquartile range and median, whiskers the 1.5 × IQR limits, and circles outlying values; no inferential test is shown. The boxes describe governorate-associated patterns within the sampled records and should not be interpreted as effects of a measured environmental variable.

Leaf relative water content (RWC) varied among the six observed governorate × age-range groups (Table 3; Figure 4), with group means ranging from 64.95% in Hebron Range I to 87.52% in Bethlehem Range II. The two Bethlehem groups had the highest means (82.85–87.52%), whereas the Hebron and Ramallah groups ranged from 64.95% to 71.07%. Within the shared Range II class, mean RWC was higher in Bethlehem (87.52 ± 5.87%) than in Hebron (71.07 ± 7.16%) and Ramallah (67.00 ± 8.20%), a difference of 16–21 percentage points that exceeded the within-group standard deviations. These governorate-associated differences may reflect variation in field conditions among governorates, including climate, soil, management practices, canopy microclimate and sampling date, but the contribution of these factors cannot be determined from the present dataset. At the orchard level, symptomatic incidence showed no clear association with leaf RWC (Spearman’s ρ = −0.100, *p* = 0.798), although this does not exclude a possible biological relationship.

Fruit pulp water content also varied among the observed governorate × age-range groups (Table 3). Within Hebron, means increased from 46.06% in Range I to 58.06% in Range II and 62.93% in Range III, while Ramallah Range II had the highest mean (64.78%). The orchard-level correlation between pulp water content and symptomatic incidence was positive (Spearman’s ρ = 0.607, *p* = 0.148) but not statistically significant; it is therefore reported as a descriptive tendency rather than evidence of a biological association.

Overall, water-status variables showed patterns that differed from symptomatic leaf-spot incidence. Leaf relative water content varied among governorates within Range II, whereas the site-level correlation with symptomatic incidence was non-significant. Fruit pulp water content had the largest positive correlation coefficient with symptomatic incidence among the measured host traits, but that association also did not reach statistical significance. These observations are descriptive and do not establish distinct causal drivers for disease and water status.

### 3.4. Photosynthetic Pigments in Relation to Suspected Cercospora Leaf-Spot Incidence

**Figure 5:**
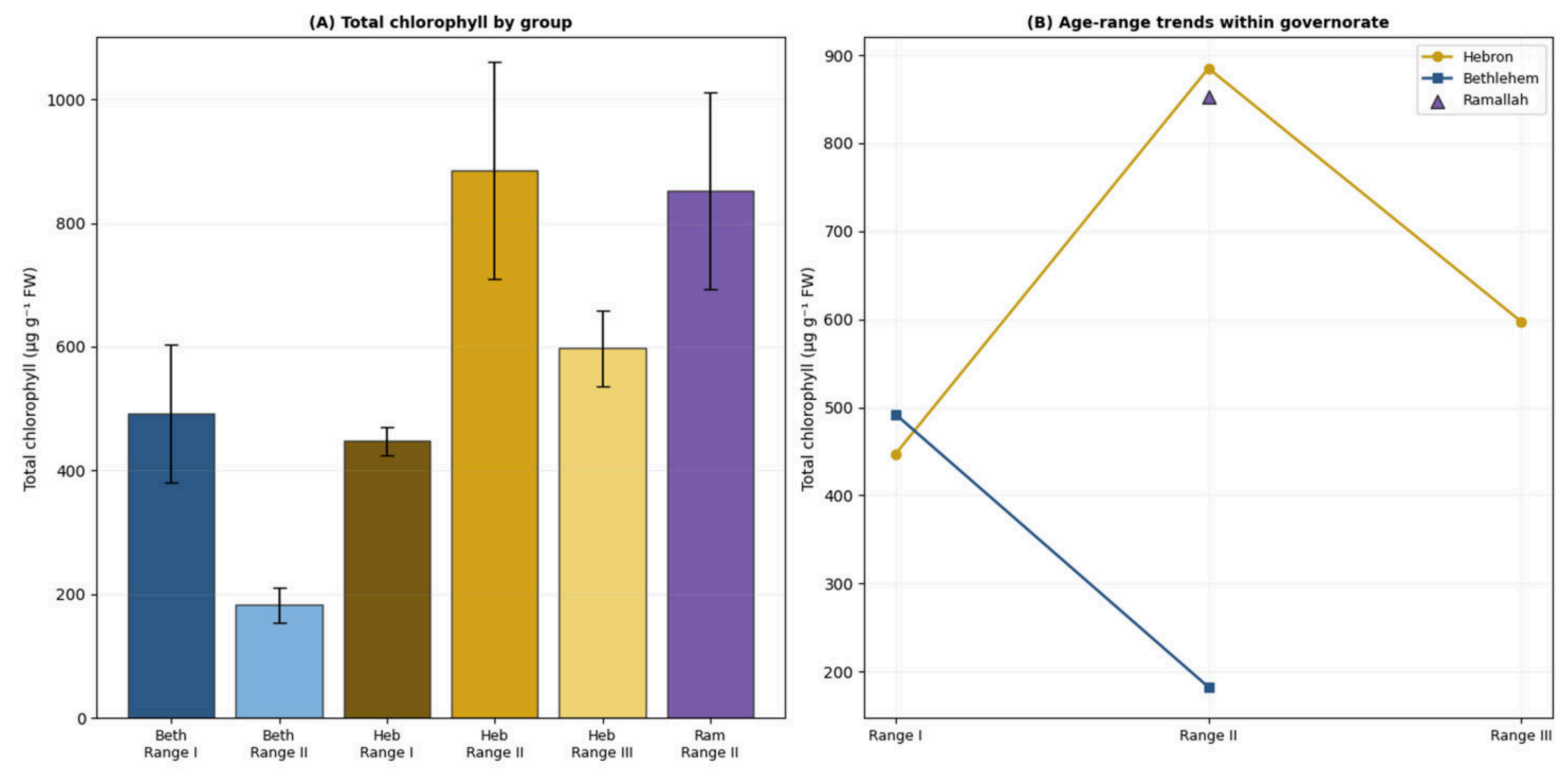
Total chlorophyll concentration by governorate × age-range group. (**A**) Means with standard-deviation error bars. (**B**) Group-mean age-range patterns within each governorate. Values are descriptive; no inferential test is shown. Concentrations were recalculated from the original absorbance readings (Section 2.5.2).

Photosynthetic pigments were described across the six governorate × age-range groups (Figure 5; Table 4). Mean total chlorophyll ranged from 182.3 µg g^−1^ FW in Bethlehem Range II to 885.1 µg g^−1^ FW in Hebron Range II, a 4.9-fold difference between the lowest and highest group means, whereas mean total carotenoids ranged from 57.6 µg g^−1^ FW in Bethlehem Range II to 159.1 µg g^−1^ FW in Hebron Range III, a narrower 2.8-fold difference. Hebron Range II and Ramallah Range II had the highest chlorophyll means, and Bethlehem Range II had the lowest means for both pigments. The carotenoid:chlorophyll ratio, calculated for each extract, was lowest in the two Range II groups with the highest total chlorophyll (Hebron and Ramallah; 0.16–0.18) and highest in Bethlehem Range II (0.32), which had the lowest chlorophyll. These comparisons rest on small and unequal numbers of extracts (three in two of the groups) and are therefore descriptive.

Within Bethlehem, total chlorophyll was lower in Range II than Range I, whereas within Hebron the largest mean occurred in Range II. Carotenoid means followed a broadly similar but not identical group ordering: the three groups with the highest total chlorophyll (Hebron Range II, Ramallah Range II and Hebron Range III) also had the highest carotenoid means, but in a different order, and Bethlehem Range II was lowest for both pigments. Across the six group means the rank correlation between the two pigments was positive but not significant (ρ = 0.771, *p* = 0.072).

Within the shared Range II age class, mean total chlorophyll was markedly lower in Bethlehem (182.3 ± 28.8 µg g^−1^ FW; *n* = 3 extracts) than in Hebron (885.1 ± 174.7 µg g^−1^ FW; *n* = 5) and Ramallah (852.4 ± 158.6 µg g^−1^ FW; *n* = 14). This describes a governorate-associated difference within one broad age stratum, based on few extracts in some groups; it is not evidence of an environmental effect or of exact control of tree age. At group level (*n* = 6), total chlorophyll had a weak, non-significant rank correlation with symptomatic incidence (ρ = 0.371, *p* = 0.468) and a positive but non-significant rank correlation with carotenoid means (ρ = 0.771, *p* = 0.072; Table S5). Given the very small number of group means, these correlations are retained only as exploratory descriptive patterns.

### 3.5. Total Phenolic Content in Relation to Suspected Leaf-Spot Incidence

**Figure 6:**
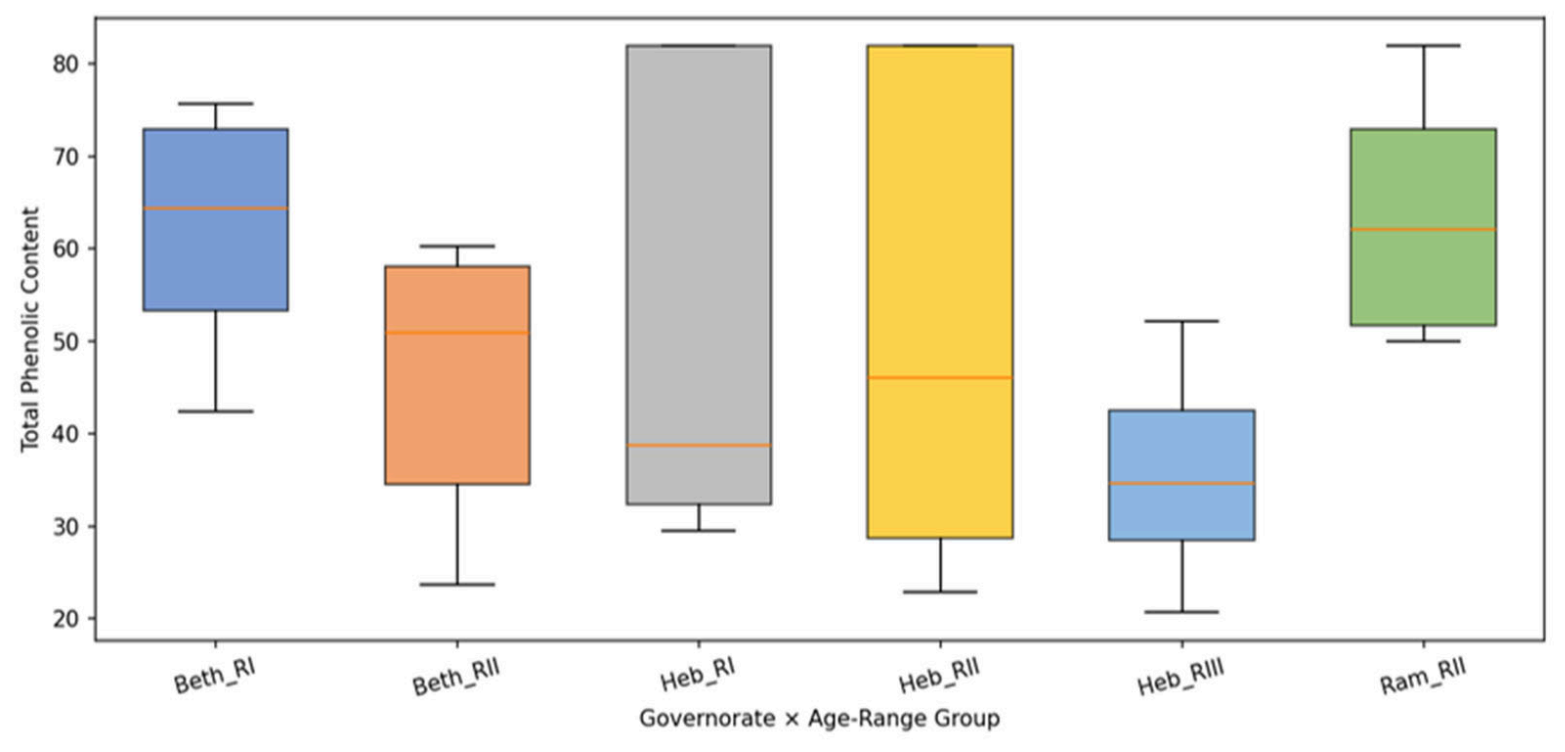
Boxplot of total phenolic content (mg GAE g^−1^ DW) by governorate × age-range group. Boxes show the interquartile range and median and whiskers the 1.5 × IQR limits; no inferential test is shown.

To examine whether symptomatic leaf-spot incidence was associated with host biochemical status, total phenolic content was determined in leaves collected from the surveyed orchards (Figure 6; Table 3). Mean total phenolic content ranged from 35.68 mg GAE g^−1^ DW in Hebron Range III to 63.86 mg GAE g^−1^ DW in Ramallah Range II, but within-group variation was large (SD 10.68–27.34 mg GAE g^−1^ DW), particularly in Hebron Ranges I and II, and the group distributions overlapped considerably. Variation in total phenolic content among groups may reflect the combined influence of tree age, location and other field factors, including environmental and orchard conditions, which cannot be separated in the present study.

Within the shared Range II age class, mean total phenolic content was 47.61 ± 13.50 mg GAE g^−1^ DW in Bethlehem, 51.88 ± 27.34 mg GAE g^−1^ DW in Hebron and 63.86 ± 12.57 mg GAE g^−1^ DW in Ramallah; given the large within-group variation, no clear governorate difference was evident. This observation cannot be taken as evidence that age accounts for the differences observed across the full set of governorate × age-range groups. At the orchard level, total phenolic content showed a positive but non-significant correlation with symptomatic incidence (Spearman’s ρ = 0.383, *p* = 0.308; Table S5). Thus, the present dataset provides no clear evidence of an association between phenolic content and symptomatic incidence, although a biological relationship cannot be excluded.

### 3.6. Variation in Fruit Characteristics in Relation to Suspected Leaf-Spot Incidence

**Figure 7:**
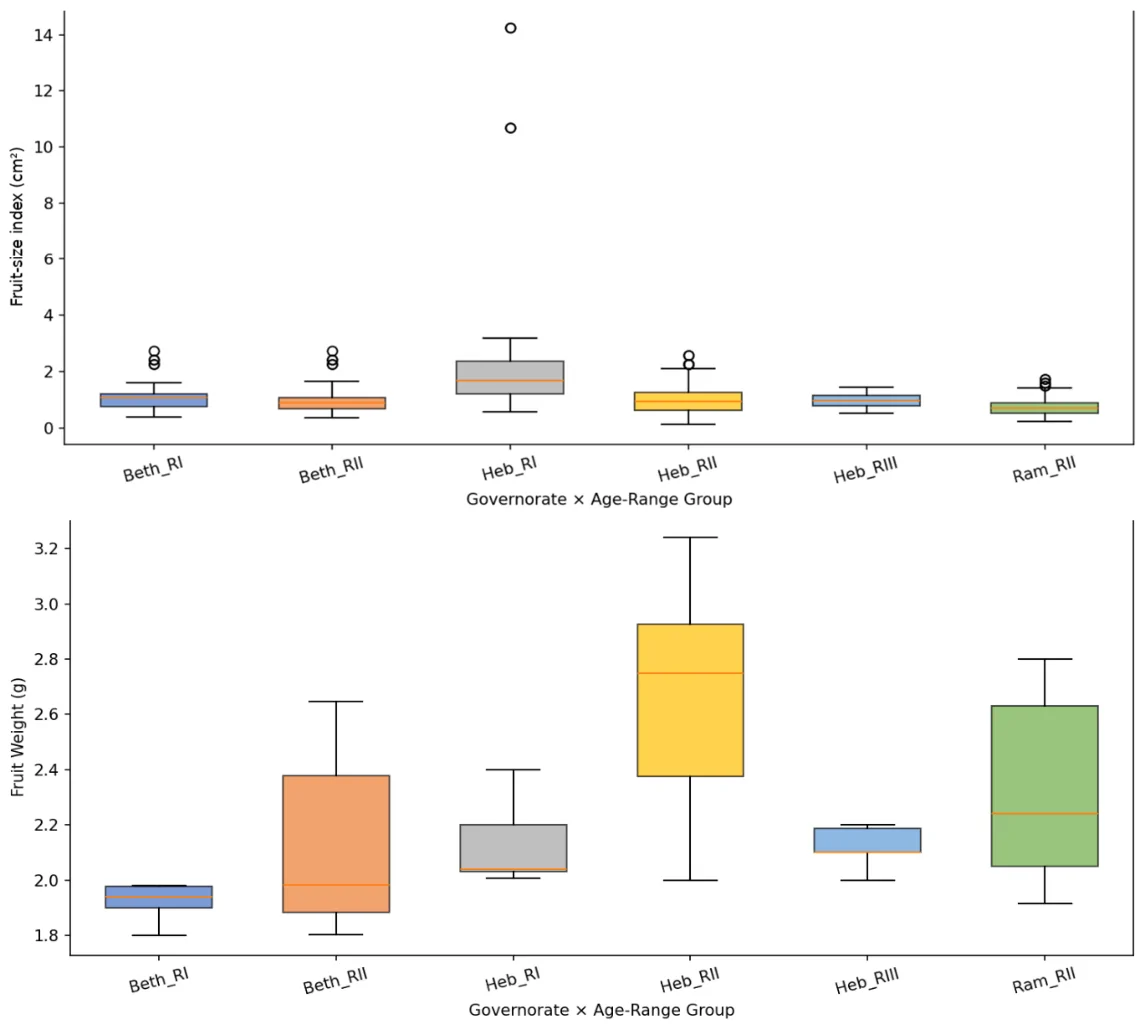
Boxplots of fruit-size index (cm^2^, **upper panel**) and fruit weight (g, **lower panel**) by governorate × age-range group. Fruit-size boxes summarize 2989 individual fruits and fruit-weight boxes 56 ten-fruit composite subsamples; because these are subsamples nested within trees and orchards, no inferential test is shown.

To assess whether disease incidence was associated with fruit characteristics, fruit weight, fruit size, pulp water content and stone-to-pulp ratio were described across the observed governorate × age-range groups (Figure 7; Table 3 and Table 5). As all surveyed orchards consisted of the same grower-identified cultivar, variation in fruit traits was not attributable to cultivar differences, although tree age and orchard conditions may have contributed. Mean fruit weight, determined from ten-fruit composite subsamples, ranged from 1.92 g in Bethlehem Range I to 2.66 g in Hebron Range II, and the mean fruit-size index, determined from 2989 individual fruits, ranged from 0.716 cm^2^ in Ramallah Range II to 1.834 cm^2^ in Hebron Range I. Because fruits and composite subsamples from the same tree are not independent biological replicates, these differences are presented descriptively; the large number of individual fruits does not increase the number of independent units, which remains limited by the number of trees and orchards. Within the shared Range II age class, mean fruit weight was higher in Hebron (2.66 ± 0.40 g) than in Ramallah (2.33 ± 0.32 g) and Bethlehem (2.12 ± 0.32 g), whereas the mean fruit-size index was lowest in Ramallah (0.716 ± 0.251 cm^2^) compared with Bethlehem (0.914 ± 0.323 cm^2^) and Hebron (0.959 ± 0.464 cm^2^), with overlapping distributions. At the orchard level, symptomatic incidence showed no clear association with fruit weight (Spearman’s ρ = 0.214, *p* = 0.645), fruit size (ρ = −0.183, *p* = 0.637) or stone-to-pulp ratio (ρ = −0.536, *p* = 0.215). Thus, the present data provide no clear evidence of an association between symptomatic incidence and the measured fruit traits under the sampled conditions, although a biological relationship cannot be excluded. Overall, fruit characteristics varied among governorate × age-range groups and among governorates within the shared Range II class, despite all orchards belonging to the same cultivar; the relative influence of climate, soil, management, microclimate, sampling and other orchard-specific factors on these differences cannot be separated from the present dataset.

### 3.7. Exploratory Integrated Relationships Among Symptomatic Leaf-Spot Incidence and Host Functional Traits

**Figure 8:**
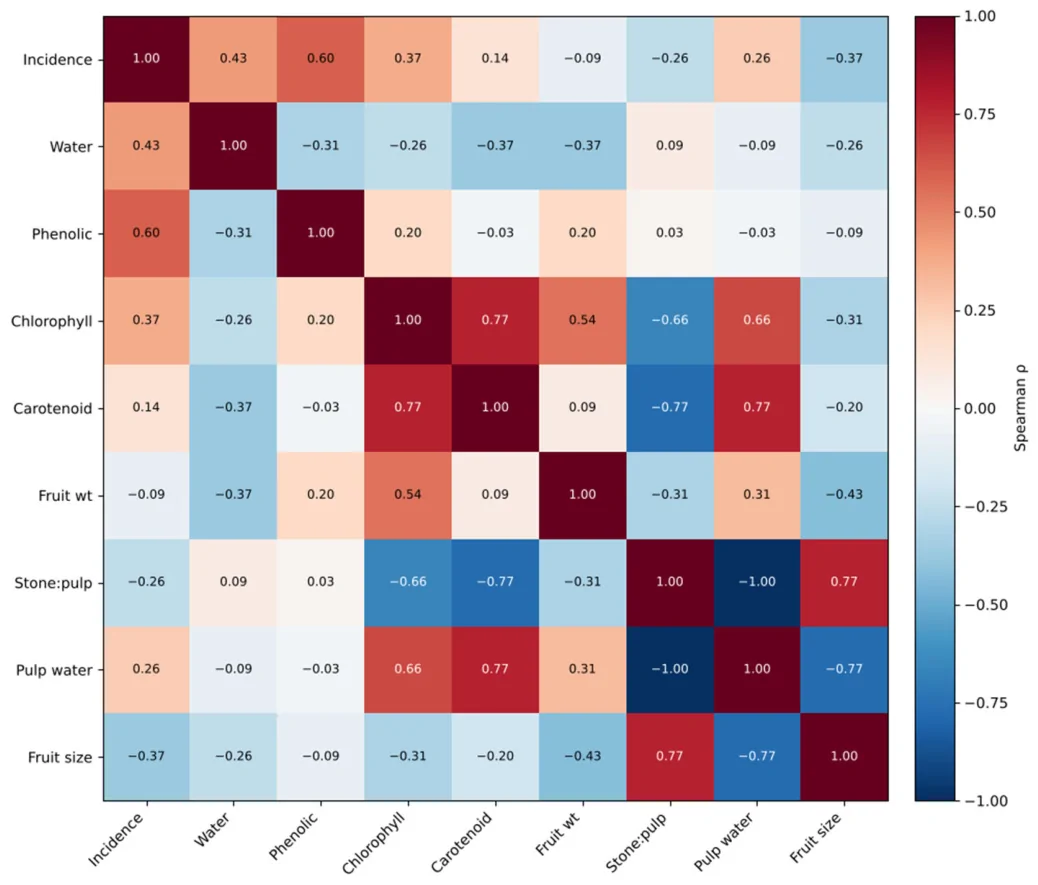
Group-level Spearman rank-order correlation matrix for the measured domains based on six governorate × age-range group means (*n* = 6). Incidence = symptomatic leaf-spot incidence. The matrix is an exploratory descriptive visualization only; with six observations, it is not used to support causal or strong inferential biological conclusions.

The correlation analyses are exploratory because the variables were aggregated at site or group level and the fully integrated matrix contains only six governorate × age-range means. Site-level correlations of symptomatic incidence with leaf relative water content (ρ = −0.100, *p* = 0.798), total phenolic content (ρ = 0.383, *p* = 0.308), pulp water content (ρ = 0.607, *p* = 0.148) and stone-to-pulp ratio (ρ = −0.536, *p* = 0.215) were non-significant. At group level, total chlorophyll and carotenoid means were positively but not significantly rank-correlated (ρ = 0.771, *p* = 0.072), and symptomatic incidence was essentially unrelated to carotenoid means (ρ = 0.143, *p* = 0.787). The full group-level matrix is shown in Figure 8. With such small *n*, all coefficients are presented as directional summaries rather than inferential evidence. Cross-domain coefficients linking fruit and leaf measurements also span adjacent seasons rather than simultaneous sampling.

**Figure 9:**
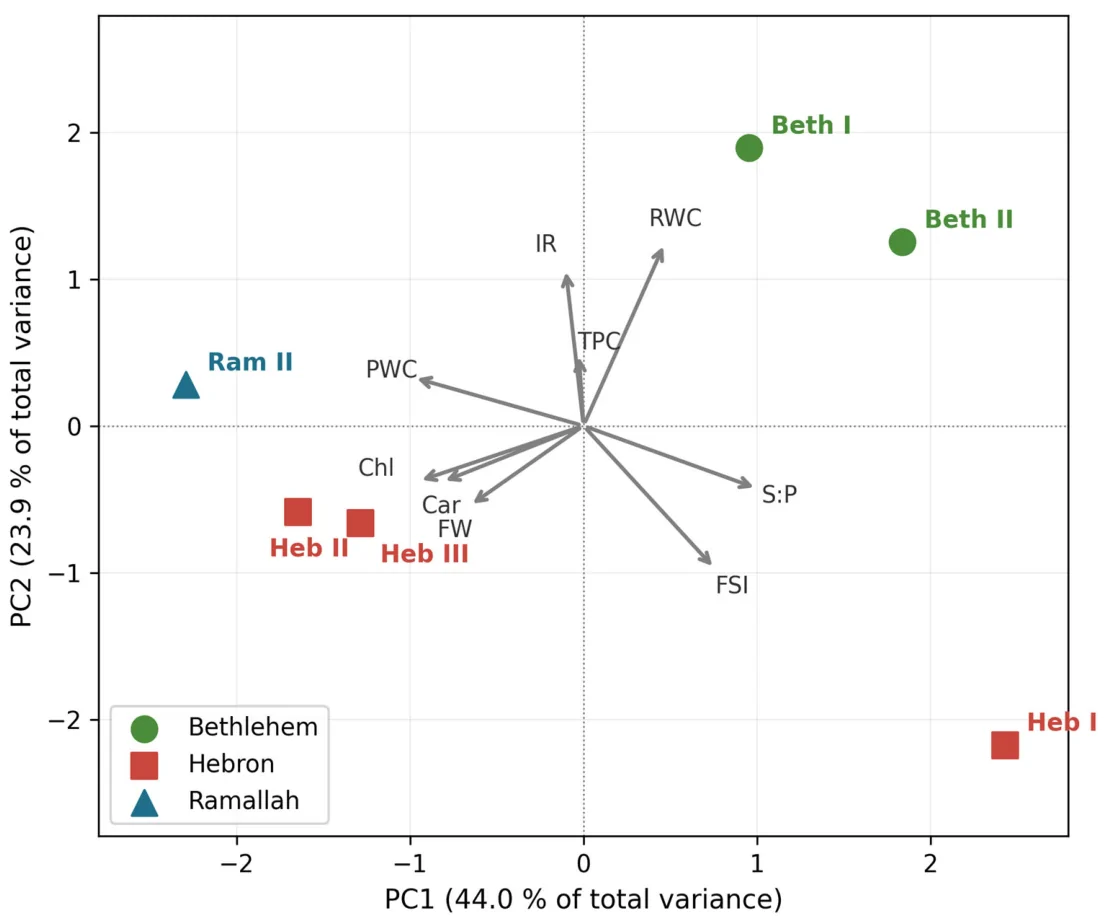
Exploratory principal component analysis biplot of six governorate × age-range group means based on nine standardized disease, physiological and pomological variables. IR = symptomatic leaf-spot incidence; RWC = leaf relative water content; TPC = total phenolic content; Chl = total chlorophyll; Car = total carotenoids; FW = fruit weight; S:P = stone-to-pulp ratio; PWC = pulp water content; FSI = fruit-size index. Because there are only six observations for nine variables, this figure is a descriptive visualization and not inferential evidence.

For an exploratory multivariate visualization, principal component analysis was applied to the standardized matrix of six group means × nine variables (Figure 9). The first two components accounted descriptively for 67.9% of variance (PC1 = 44.0%, PC2 = 23.9%). PC1 had its largest loadings on stone-to-pulp ratio, pulp water content, total chlorophyll, total carotenoids and fruit-size index, while symptomatic incidence had a near-zero loading (−0.05); PC2 had larger loadings for leaf relative water content (+0.57), symptomatic incidence (+0.49) and fruit-size index (−0.45). With only six observations for nine variables, these loadings are unstable and are not treated as inferential evidence.

The biplot visually separated the two Bethlehem groups from Hebron Range II and Ramallah Range II, while Hebron Range I occupied a different position from the younger Hebron groups. Descriptively, the arrangement reflects the joint governorate × age composition of the six group means, and symptomatic incidence contributes little to PC1. Given *n* = 6 and the unbalanced design, the ordination cannot establish that disease is biologically orthogonal to host physiology or that age or governorate drives the observed separation.

In summary, mean symptomatic leaf-spot incidence varied among the six groups, mainly because of the higher mean in Bethlehem Range I, but was similar among governorates within the shared Range II age class. Within Range II, mean leaf relative water content, total chlorophyll and fruit weight differed among governorates, mean fruit-size index was lowest in Ramallah with overlapping distributions, and total phenolic content overlapped widely. These descriptive contrasts characterize the sampled orchards but do not separate independent age and governorate effects or identify specific environmental causes. Correlations and PCA are retained only as exploratory summaries. Table 5 presents a cautious synthesis of these descriptive patterns.

Overall, symptomatic incidence and host-trait measurements displayed different descriptive patterns across the sampled orchards. Mean incidence varied among the six groups but was similar among governorates within Range II, whereas several host traits differed among governorates within that class. These observations justify explicit, balanced age stratification in future sampling, but they neither establish a tree-age effect nor attribute governorate-associated differences to measured environmental drivers.

## 4. Discussion

### 4.1. Suspected Cercospora Leaf-Spot Incidence Across Governorate × Age-Range Groups and Within the Shared Age Class

Mean symptomatic incidence varied among the six sampled governorate × age-range groups, from 9.29% in Hebron Range I to 14.11% in Bethlehem Range I, but the branch-level distributions overlapped widely, and the variation did not follow a monotonic age pattern: within Hebron, the intermediate Range II group had a higher mean than Ranges I and III, whereas in Bethlehem Range I exceeded Range II. Within Range II, the only broad age class represented in all three governorates, mean incidence was similar among governorates (9.95–11.02%). These two descriptive observations are reported side by side, but the design cannot determine why they differ. The absence of a clear governorate difference within Range II does not show that orchard age structure caused or contributed to the six-group pattern; it shows only that no marked governorate difference was observed within one broad 25–50-year class. Because age classes were unevenly represented among governorates and were broad, exact tree ages were unavailable, each group comprised few orchards and sampling dates differed, the six-group pattern cannot be attributed to tree age, governorate, orchard-specific conditions or their combination.

Where older orchards do experience greater disease pressure, several orchard-level mechanisms are biologically plausible. Mature trees can develop larger and denser canopies, creating humid, poorly ventilated microenvironments that favor conidial germination and prolong leaf wetness. Long-lived orchards may also maintain inoculum over repeated seasons, given the long incubation period and year-round inoculum production reported for *P. cladosporioides* [8,17]; survival of inoculum in infected foliage has also been documented for *V. oleaginea* in the comparable peacock spot pathosystem [12]. Tree developmental stage may additionally influence leaf turnover, light interception, ventilation, tree vigor and the duration of leaf exposure to environmental conditions [1,2,6]. These mechanisms offer hypotheses for the elevated incidence observed in Bethlehem Range I, but the present observational design did not measure canopy density, leaf wetness or inoculum load directly and therefore cannot attribute this pattern to tree age or to any single mechanism. Moreover, the other old-orchard group (Hebron Range I) had the lowest mean incidence, so the elevated incidence in Bethlehem Range I cannot be generalized to older orchards.

Tree age may also influence host susceptibility through physiological and structural changes. Older olive trees retain leaves for longer periods, thereby increasing the exposure time of susceptible tissues to airborne conidia. Furthermore, age-related modifications in canopy architecture, resource allocation, and vegetative renewal may indirectly favor disease establishment by reducing the replacement of infected foliage. It should be noted, however, that the relationship between leaf age and infection is not necessarily monotonic. In the comparable peacock spot pathosystem, controlled experiments with *V. oleaginea* have shown that conidial germination and infection efficiency decline with increasing individual leaf age [36,37]; whether the same applies to *P. cladosporioides* has not been established. If it does, elevated incidence in particular old orchards, such as Bethlehem Range I, would be more plausibly attributable to orchard-level inoculum accumulation and canopy microclimate than to any intrinsic increase in the susceptibility of individual leaves. This distinction between tree age and leaf age deserves explicit attention in future work, and could be tested directly by scoring cohorts of leaves of known emergence date within trees of contrasting age, an approach used to study leaf-age effects for *V. oleaginea* [38].

Within the shared Range II age class, mean incidence was similar among the three governorates despite their different rainfall and humidity regimes. This observation does not identify the cause of the six-group pattern; it indicates only that, in the sampled orchards, governorate differences in mean incidence were small within one broad age class. While temperature, rainfall and relative humidity regulate infection by *P. cladosporioides* [8,17], their effects may interact with orchard management practices, local microclimatic conditions, and host status. Soil characteristics, water availability, canopy management, altitude, exposure and local microclimate may all contribute to differences in host condition and disease expression [1,2,39,40]. The station-level climatic records for January–September 2024 correspond to the season preceding the fruit harvest, but they are governorate-level records that were not measured in the orchards and do not cover the October 2024–April 2025 period preceding leaf sampling; they should therefore not be interpreted as direct measurements of the environmental conditions prevailing during disease development. The relatively narrow range of incidence observed across the surveyed orchards may also be biologically informative. Cercospora leaf spot can persist and develop under environmental conditions that vary among olive-growing areas, and its epidemiology is influenced by inoculum production and infection timing throughout the year [17]. Consequently, differences among orchards may become more apparent when tree developmental stage, canopy environment and seasonal conditions are considered together rather than through geographic location alone.

Overall, the unbalanced age structure of the sampled orchards indicates that tree age should be explicitly recorded and balanced in surveillance and comparative epidemiological studies of suspected Cercospora leaf spot. The present data do not support the assumption that age alone predicts disease pressure: the highest mean incidence occurred in one old-orchard group (Bethlehem Range I), whereas the other old-orchard group (Hebron Range I) had the lowest mean. More balanced sampling across age classes and locations, combined with exact tree ages, complete tree and orchard identifiers and repeated seasonal observations, would provide a stronger basis for determining the consistency and biological significance of these patterns.

### 4.2. Governorate-Associated Variation in Host Water Status and Its Relationship to Suspected Cercospora Leaf-Spot Incidence

Water status is a fundamental determinant of plant growth, physiological performance, and disease response. In the present study, leaf relative water content (RWC) and fruit pulp water content exhibited response patterns that differed from those of suspected Cercospora leaf-spot incidence. Within the common 25–50-year age range, mean leaf RWC was 16–21 percentage points higher in Bethlehem than in the corresponding Hebron and Ramallah groups. This observation indicates that trees belonging to a broadly comparable age class may nevertheless differ substantially in their water status according to growing location or sampling date. Such variation is consistent with the strong influence of environmental conditions and orchard water availability on olive physiological performance [1,2,39,40]. A related question is whether greater water availability itself favours disease, since prolonged leaf wetness and high atmospheric humidity are prerequisites for conidial germination and penetration in olive foliar pathogens, as shown for *V. oleaginea* in the comparable peacock spot pathosystem [12,37]. However, the present data do not indicate a simple relationship between host water status and symptomatic disease incidence. Within the age-class-restricted Range II comparison, mean symptomatic incidence was similar among the three governorates, whereas mean leaf RWC differed markedly. The higher RWC observed in Bethlehem therefore did not correspond to higher disease incidence. This divergence suggests that leaf hydration alone does not explain the spatial pattern of symptomatic disease observed in the present survey. Moreover, total rainfall is only an indirect measure of the moisture conditions governing infection, which depend on rainfall distribution, dew formation, canopy density and leaf-wetness duration; accordingly, weather-based forecasting models developed for peacock spot (*V. oleaginea*) are driven by temperature and wetness conditions rather than by rainfall totals alone [41].

The differences in mean leaf relative water content observed among governorates within the broad Range II class suggest that environmental and orchard-level factors may play an important role in determining the water status of olive trees. Olive is recognized as one of the most drought-adapted fruit tree species, possessing morphological and physiological mechanisms that allow it to maintain tissue hydration under variable environmental conditions [1,2]. Consequently, differences in soil characteristics, soil water availability, altitude, atmospheric demand and local microclimate may contribute to the observed variation in leaf hydration [39,40]. Because these factors were not measured directly in the present survey, their relative contribution to the observed differences in RWC remains uncertain. The climatic records available for the study describe the 2024 growing season at governorate level, but because they were not measured in the orchards and do not extend to the spring 2025 leaf-sampling period, they should be considered contextual rather than explanatory.

Leaf relative water content showed no clear orchard-level correlation with symptomatic incidence (ρ = −0.100, *p* = 0.798; Section 3.3), so higher incidence was not associated with reduced leaf hydration in this dataset. Disease development represents a cumulative process, whereas RWC is a physiological measurement reflecting the water status of the plant at the time of sampling. The absence of a clear relationship therefore does not exclude interactions occurring earlier in the season or involving soil moisture, canopy humidity or leaf-wetness duration.

Fruit pulp water content exhibited a different behaviour. Although its correlation with disease incidence was not statistically significant, it showed the strongest positive association among the physiological and pomological traits evaluated. This trend is potentially interesting because fruit water status may integrate environmental and physiological conditions over a longer developmental period than a single measurement of leaf hydration. Fruit growth extends over several months and reflects the balance between water uptake, carbon assimilation, and assimilate partitioning [1,2,42]. It must be stressed, however, that the fruit and leaf campaigns were conducted in adjacent seasons rather than concurrently. Therefore, this association should be considered an exploratory field pattern rather than evidence of a simultaneous disease–water relationship.

Collectively, these findings indicate that host water relations and suspected Cercospora leaf-spot incidence showed largely different patterns across the surveyed orchards. The governorate-associated differences in mean RWC, together with the absence of a clear incidence–RWC relationship, suggest that factors beyond current leaf hydration contribute to disease expression. Future studies combining disease observations with simultaneous measurements of leaf water status, soil moisture, relative humidity and leaf wetness at the tree and orchard levels would be particularly valuable for determining whether water relations influence infection and disease progression.

### 4.3. Photosynthetic Pigments and Phenolic Compounds in Relation to Symptomatic Leaf-Spot Incidence

Photosynthetic pigments and phenolic compounds are important indicators of plant physiological status and defence responses [19,20]. Changes in chlorophyll concentration and photosynthetic performance have been reported in molecularly confirmed foliar pathosystems following pathogen infection [22]. In the present survey, the observed variation in pigment and phenolic traits provides complementary information on the physiological heterogeneity of olive trees across governorates and age ranges. Although the causal species was not molecularly confirmed and disease was quantified as symptomatic incidence rather than lesion-area severity, these measurements remain valuable for characterizing host responses associated with the observed disease pattern.

Mean total chlorophyll varied among the sampled groups and among governorates within Range II, indicating spatial variation in this photosynthetic trait even among trees within the same broad age class, although some groups were represented by only three extracts. However, no clear group-level correlation with symptomatic incidence was found (ρ = 0.371, *p* = 0.468). This indicates that the spatial pattern of chlorophyll concentration did not simply follow the proportion of symptomatic leaves. Several environmental and orchard-level factors, including water availability, temperature, solar exposure, nitrogen status, soil properties, canopy structure and sampling date, may contribute to this variation [1,2,23,39]. Their individual and combined effects could not be separated in the present survey, but their potential contribution does not diminish the observed governorate-associated differences in pigment status.

One possible biological explanation is that localized foliar lesions may not produce a proportional reduction in whole-leaf pigment concentration when substantial portions of the leaf remain asymptomatic and photosynthetically functional [19,43]. This possibility is particularly relevant because symptomatic incidence represents the proportion of leaves exhibiting symptoms, whereas lesion-area severity provides a measure of the extent of tissue affected within individual leaves. The absence of a clear relationship between incidence and total chlorophyll therefore suggests that symptom occurrence alone may not adequately capture the physiological impact of the disease. Direct measurements of lesion severity, chlorophyll fluorescence and gas exchange would be useful to further investigate this relationship.

Carotenoid group means varied less among groups than total chlorophyll (a 2.8-fold versus a 4.9-fold range between the lowest and highest group means) and followed a broadly similar but not identical group ordering, yielding a positive, non-significant rank correlation across the six group means (ρ = 0.771, *p* = 0.072). Correspondingly, the extract-level carotenoid:chlorophyll ratio was lowest in the chlorophyll-rich Hebron Range II and Ramallah Range II groups and highest in the chlorophyll-poor Bethlehem Range II group, a pattern consistent with a relatively conserved carotenoid pool against a more variable chlorophyll pool. Given the small number of group means (*n* = 6), the small pigment sample, and the absence of any association between carotenoids and symptomatic incidence (ρ = 0.143, *p* = 0.787), this result is appropriately considered a descriptive indication of partially coordinated variation rather than evidence of a specific photoprotective mechanism. Nevertheless, the contrasting variability of the two pigment classes provides an interesting physiological pattern that merits confirmation using independent tree-level measurements.

Total phenolic content exhibited a different response pattern. Phenolic compounds have recognized structural and biochemical roles in plant defence and may contribute to resistance against fungal pathogens through direct antimicrobial activity, antioxidant functions and modulation of defence responses [20,21]. In olive, phenolic metabolism has also been associated with defence against peacock spot caused by *V. oleaginea* [24]. Mean total phenolic content varied among the six groups, but within Range II the governorate means overlapped widely, and no clear orchard-level correlation was found between phenolic concentration and symptomatic incidence. Phenolic concentration is influenced by numerous factors, including developmental stage, environmental conditions and plant physiological status [20,21,24], and its relationship with disease is therefore unlikely to be captured adequately by a single total-phenolic measurement. The observed variation across age ranges may therefore reflect developmental and environmental differences rather than a direct response to pathogen infection.

Taken together, these findings indicate that photosynthetic pigments and total phenolics showed different patterns across the sampled orchards. The pigment and phenolic results reveal substantial biochemical and physiological differentiation among the surveyed orchards, but these traits did not simply follow the distribution of symptomatic disease. This divergence is informative because variation in host physiological and biochemical status was broader than the observed disease-incidence pattern. Under the natural field conditions of the present survey, neither pigment nor total phenolic concentration emerged as a reliable predictor of symptomatic incidence. Future studies combining disease incidence with lesion severity, chlorophyll fluorescence, gas exchange and specific phenolic profiles could provide a more detailed understanding of how olive physiological responses relate to suspected Cercospora infection.

### 4.4. Governorate-Associated Variation in Fruit Development and Its Relationship to Suspected Leaf-Spot Incidence

Fruit development represents the cumulative outcome of the physiological processes occurring throughout the growing season and is therefore influenced by both environmental conditions and plant health. Fruit characteristics therefore provide a complementary perspective on orchard variability because fruit development integrates environmental conditions and tree physiological performance over an extended period. Foliar diseases such as cercosporiosis have the potential to affect fruit growth indirectly by reducing the photosynthetically active leaf area available for carbon assimilation, thereby limiting the supply of assimilates required for fruit development [19,43]; experimental manipulation of the leaf-to-fruit ratio in olive confirms that photosynthate availability constrains fruit growth and ripening [28]. Consequently, alterations in fruit weight, fruit size and fruit composition are frequently considered indicators of the longer-term physiological status and productive performance of the tree. In the present study, mean fruit weight and fruit-size index varied among the surveyed groups; within the common Range II age class, fruit weight differed among governorates and fruit-size index was lowest in Ramallah. These traits therefore showed spatial variation in fruit development even among trees within the same broad age class. Olive fruit development is known to be highly responsive to environmental factors, particularly water availability during fruit enlargement, which directly affects cell expansion and final fruit size [1,2,42]. This is consistent with the broader evidence that, although fruit fresh weight is strongly heritable, the growing environment substantially modifies fruit expression, water status and phenolic composition across olive-growing regions [25,26,27]. Water availability, temperature, soil characteristics and orchard management may all contribute to these differences, although their relative effects were not directly evaluated in the present study.

Although no statistically significant relationships were detected between symptomatic disease incidence and the evaluated pomological traits, fruit pulp water content exhibited the strongest positive association with symptomatic incidence among the physiological and pomological variables measured. Likewise, the stone-to-pulp ratio tended to decrease as disease incidence increased. However, neither association was statistically significant, and these trends should therefore be interpreted as exploratory rather than as evidence of a direct disease effect. Nevertheless, they suggest that fruit composition may integrate cumulative physiological conditions more effectively than fruit size or fruit weight alone. Unlike leaf measurements, which represent the physiological status at a specific sampling date, fruit composition reflects the balance between carbon assimilation, water relations and assimilate allocation throughout the growing season. The absence of clear correlations between disease incidence and most fruit characteristics indicates that the observed variation in symptomatic incidence was not accompanied by a detectable relationship with fruit development in the present dataset. Similar observations have been reported for perennial fruit crops, where moderate levels of foliar disease may have limited effects on yield components because healthy foliage compensates for the loss of photosynthetically active tissue [19]. Nevertheless, repeated infections over successive years may gradually affect tree vigour and productivity, particularly where disease pressure is sustained [11,19,28,43]. The potential impact of chronic foliar disease may therefore become more apparent when disease severity and productivity are monitored simultaneously over several seasons. The temporal relationship between the two datasets is particularly relevant. Fruit measurements were obtained during the preceding harvest, whereas leaf symptoms were assessed during the following spring. Fruit weight and size therefore represent the outcome of environmental and physiological conditions accumulated over the fruit-development period, while symptomatic incidence reflects disease expression during a subsequent campaign. A strong contemporaneous correlation should not necessarily be expected under these circumstances, and the observed associations should consequently be interpreted as exploratory field patterns rather than direct evidence of disease effects on fruit development. The fruit results therefore extend the epidemiological analysis beyond disease incidence and indicate that productive traits varied geographically even where mean incidence was similar within Range II. This distinction underlines the importance of considering disease pressure together with the broader physiological and productive context of olive orchards. Multi-season monitoring combining disease incidence and severity with fruit growth, yield and physiological measurements would be particularly valuable for determining whether the exploratory relationships observed here become more evident under sustained disease pressure.

### 4.5. Integrating the Epidemiological and Physiological Responses of Olive Trees

The present study provides a descriptive field assessment of suspected Cercospora leaf-spot incidence together with host physiological performance and fruit characteristics under natural field conditions. The combined analysis provides an integrated view of disease incidence, host physiology, biochemical status and fruit development, revealing that these response domains did not exhibit identical spatial patterns. Although olive foliar disease is traditionally regarded as a constraint capable of impairing photosynthetic activity and reducing tree productivity, the present observations indicate that symptomatic incidence did not track the observed variability in the physiological and pomological traits of the sampled trees.

The exploratory PCA is consistent with this description but provides no independent evidence for it. The first two components accounted for 67.9% of the variance among the six group means (PC1 = 44.0%, PC2 = 23.9%), and symptomatic incidence had a near-zero loading on PC1 (−0.05). With six observations and nine variables, the ordination is unstable and is regarded here only as a visualization of the six aggregated group means; it cannot establish that disease incidence is independent of the main physiological gradient or reinforce other conclusions. Visually, the two Bethlehem groups were positioned close together, Hebron Range II and Ramallah Range II lay close to each other despite belonging to different governorates, and Hebron Range I was separated from the younger Hebron groups, reflecting the joint governorate × age composition of the group means. The ordination is therefore useful mainly for generating hypotheses for studies with independent tree- and orchard-level replication.

A central descriptive outcome of this study is that mean symptomatic incidence varied among the six sampled groups but was similar among governorates within the shared Range II class, whereas several physiological and fruit traits differed among governorates within that class. Because governorate and age were not crossed, age classes were broad, exact ages were unavailable and each group comprised few orchards, the design cannot determine why the six-group and Range II incidence comparisons differed, nor separate age, governorate, orchard and sampling-date influences. The contrast nevertheless indicates that symptomatic incidence and host performance did not follow the same patterns in the sampled orchards. No monotonic age pattern was observed: within Hebron, mean incidence was highest in Range II rather than Range I. Tree age should therefore be treated as an essential design variable in future surveys, because its possible influence may interact with orchard-specific canopy, inoculum and microclimatic conditions. Among the evaluated variables, fruit pulp water content showed the largest positive correlation coefficient with symptomatic incidence, although this orchard-level relationship was not statistically significant. This observation is potentially informative because fruit water status may integrate cumulative differences in orchard conditions that are not reflected by a single spring measurement of leaf RWC. Because fruit development depends on the long-term balance between photosynthesis, water relations, assimilate allocation and environmental conditions, fruit composition may provide a more integrative assessment of tree performance than individual physiological measurements. However, because fruit and disease measurements were obtained during adjacent rather than concurrent campaigns, this relationship should be interpreted as an exploratory field pattern rather than evidence of a direct disease effect.

Overall, the present study supports a multifactorial interpretation of olive orchard variability, in which tree developmental stage, local environmental conditions, orchard characteristics, host physiological status and disease pressure may interact. The strength of the present survey lies in bringing epidemiological, physiological, biochemical and pomological information together under natural field conditions. Rather than identifying a single determinant of disease incidence, the study highlights the multidimensional context in which suspected Cercospora leaf spot occurs and provides a field-based framework for more targeted investigations. Future balanced, multi-season studies combining disease incidence and severity with contemporaneous environmental and physiological measurements will be particularly valuable for disentangling age, site and microclimate effects.

### 4.6. Limitations and Future Perspectives

Several aspects of the study design should be considered when interpreting the findings. First, age and governorate were not fully factorially crossed: Ramallah included only Range II, Bethlehem included two age classes, and Hebron included three. The age strata also differed substantially in width (approximately 5, 25 and 50 years), and exact individual-tree ages were unavailable. These features limit direct separation of age-related and geographic variation and highlight the importance of more balanced age representation in future surveys.

Second, leaf sampling dates differed among governorates over approximately six weeks during spring 2025. Seasonal phenology and sampling date may therefore contribute to some of the observed physiological differences. Third, branches and individual fruits were nested within trees, and trees were nested within orchards. Complete tree–orchard identifiers were not available for all record-level observations; consequently, mixed-effects models could not be reconstructed for the entire branch- and fruit-level datasets. For this reason, group comparisons were presented descriptively, without inferential tests on branch-, fruit- or extract-level records. In addition, each governorate × age-range group was represented by only one or a few of the nine orchards, so orchard-to-orchard variation cannot be separated from group differences.

Fourth, the integrated correlations and PCA were based on a small number of aggregated observations, including six group means. These analyses are consequently exploratory; the PCA in particular is unstable with six observations and nine variables and serves only as a visualization of the group means. Both analyses nevertheless remain useful for describing the structure of the observed dataset and for identifying relationships worthy of testing with larger independent datasets. Fifth, fruit and spring leaf measurements were obtained in adjacent campaigns rather than concurrently, which limits direct temporal integration between fruit development and subsequent leaf responses.

Sixth, the suspected pathogen was characterized using symptomatology and conidiophore/conidial morphology observed directly on lesions, without molecular confirmation; the colony appearance of the isolates is not species-diagnostic, and neither the isolates nor the lesions were identified by sequencing. Typical peacock-spot lesions attributable to *Venturia oleaginea* were operationally excluded, although mixed infections cannot be completely ruled out without lesion-level molecular diagnosis. Seventh, the pigment dataset comprised 38 extracts unevenly distributed among groups (three extracts in two of the groups), and two extracts returned physically impossible chlorophyll *b* or carotenoid estimates that had to be excluded; pigment comparisons therefore rest on a small sample and should be confirmed with larger, tree-level replication. Eighth, the available phenolic-extraction record does not establish whether an additional 24-h cold hold was applied uniformly across all samples, introducing a procedural uncertainty that should be addressed in future work. Ninth, disease was quantified as symptomatic leaf incidence rather than lesion-area severity; incidence and severity consequently represent complementary but distinct measures of disease expression. Finally, the study represents one fruit harvest and one subsequent spring leaf campaign.

These limitations define the scope of inference but do not diminish the value of the field observations. Rather, they identify specific methodological improvements that can build directly on the present baseline. Future studies should employ balanced age × governorate sampling with narrower and preferably exact age classes, retain complete tree and orchard identifiers for hierarchical modelling, synchronize sampling within comparable phenological windows, and collect contemporaneous measurements of weather, leaf wetness, soil conditions and orchard management. Fruit and leaf measurements should preferably be paired within the same season, while raw spectrophotometric data and fully standardized extraction records should be preserved. Molecular confirmation of the causal agent, including assessment of possible mixed infections through ITS/multilocus approaches or targeted quantitative PCR, would further strengthen disease attribution. Multi-season monitoring combining disease incidence and severity with physiological, biochemical and yield-related traits would ultimately provide the strongest basis for determining how disease pressure interacts with tree age and orchard environment.

## 5. Conclusions

This descriptive survey showed that mean suspected Cercospora leaf-spot incidence varied among the six sampled governorate × age-range groups, from 9.29% in Hebron Range I to 14.11% in Bethlehem Range I, with widely overlapping branch-level distributions. Within the 25–50-year age class shared by all three governorates, mean incidence was similar among governorates (9.95–11.02%). The design cannot determine why these two comparisons produced different outcomes, and it does not show that tree age, governorate or orchard age structure caused the six-group pattern. Incidence also did not change monotonically with age range; within Hebron, the intermediate age range had the highest mean. In contrast, within the shared age class, mean leaf relative water content, total chlorophyll and fruit weight differed among governorates, mean fruit-size index was lowest in Ramallah, and total phenolic content overlapped widely. Exploratory orchard- and group-level correlations showed no clear association between symptomatic leaf-spot incidence and leaf water content, total chlorophyll, phenolic compounds, fruit weight or fruit size; the largest, although non-significant, coefficients involved pulp water content (ρ = 0.607) and stone-to-pulp ratio (ρ = −0.536). Total chlorophyll and carotenoid group means were positively but not significantly rank-correlated (ρ = 0.771, *p* = 0.072), and the principal component analysis served only as a visualization of the six group means. Overall, symptomatic incidence, host physiological status and fruit characteristics showed different descriptive patterns in the sampled orchards. The novelty of this work lies in documenting suspected Cercospora leaf-spot incidence together with physiological, biochemical and productive traits in rain-fed Palestinian olive orchards. Future balanced, age-resolved surveys with complete tree and orchard identifiers, molecular pathogen identification, disease-severity assessment and contemporaneous environmental measurements are needed to clarify the factors underlying disease variability.

## Figures and Tables

**Table 1:** Monthly climate characteristics of the three studied governorates, January–September 2024 (the growing season preceding the October–November 2024 fruit harvest): mean air temperature (T, °C), mean relative humidity (RH, %) and total rainfall (R, mm).

Month (2024)	Ramallah			Hebron			Bethlehem		
	T (°C)	RH (%)	R (mm)	T (°C)	RH (%)	R (mm)	T (°C)	RH (%)	R (mm)
Jan	13.5	80	265.8	13.0	84	204.0	13.25	82	235.5
Feb	13.3	79	95.7	12.9	86	54.6	13.10	82.5	60.5
Mar	17.2	66	61.0	17.0	75	24.3	17.10	70.5	55.5
Apr	22.9	62	15.7	22.8	69	16.9	22.85	65.5	45.0
May	24.0	62	11.5	24.3	68	5.0	24.15	65.0	3.5
Jun	30.9	47	0.0	31.3	50	0.0	31.10	48.5	0.0
Jul	30.0	60	0.0	30.4	65	0.0	30.20	62.5	0.0
Aug	29.3	67	0.0	29.9	71	0.0	29.60	69.0	0.0
Sep	27.2	71	2.2	26.5	82	1.4	26.85	76.5	11.5
Jan–Sep mean/total	23.1	66.0	451.9	23.1	72.2	306.2	23.1	69.1	411.5

T = monthly mean air temperature; RH = monthly mean relative humidity; R = monthly total rainfall. The final row gives the January–September mean for T and RH and cumulative total for R. Source: Palestinian Meteorological Directorate/Palestinian Central Bureau of Statistics [31]. Bethlehem T and RH values were reconstructed by interpolation between the Ramallah and Hebron stations. Reference mean annual rainfall is 615.2 mm (Ramallah and Al-Bireh), 595.9 mm (Hebron) and 518.4 mm (Bethlehem) [31].

**Table 2:** Sampling design and number of measurement records/subsamples per governorate × age-range group and analytical domain; counts are not equivalent to independent experimental units.

Governorate × Age-Range Group	Governorate	Age (yr)	IR	RWC	TPC	Chl	Fruit	Fruit Size
Bethlehem_RangeI	Bethlehem	50–100	46	5	4	8	5	250
Bethlehem_RangeII	Bethlehem	25–50	74	10	9	3	10	449
Hebron_RangeI	Hebron	50–100	77	5	5	3	5	500
Hebron_RangeII	Hebron	25–50	76	18	8	5	16	740
Hebron_RangeIII	Hebron	15–20	40	5	10	5	5	300
Ramallah_RangeII	Ramallah	25–50	116	15	12	14	15	750
Total	—	—	429	58	48	38	56	2989

All records were obtained from the 45 tagged trees (five per orchard) in the nine orchards. IR = symptomatic leaf-spot incidence records at branch level; RWC = leaf relative water content samples; TPC = total phenolic content samples; Chl = photosynthetic-pigment samples; Fruit = ten-fruit composite subsamples used for fruit weight, stone-to-pulp ratio and pulp water content; Fruit size = individual fruits measured. Branches and fruits are nested within trees, and trees are nested within orchards; therefore, the 429 branch records and 2989 individual-fruit measurements must not be interpreted as 429 and 2989 independent biological replicates.

**Table 3:** Group means with standard deviations in parentheses for the nine analytical domains across the six governorate × age-range groups.

Domain	Beth I	Beth II	Heb I	Heb II	Heb III	Ram II
Symptomatic leaf-spot incidence (%)	14.11 (5.73)	9.95 (5.07)	9.29 (4.11)	10.89 (4.67)	9.93 (4.39)	11.02 (5.70)
Leaf relative water content (%)	82.85 (7.15)	87.52 (5.87)	64.95 (6.27)	71.07 (7.16)	68.32 (12.00)	67.00 (8.20)
Total phenolic content (mg GAE g^−1^ DW)	61.75 (15.25)	47.61 (13.50)	52.94 (26.70)	51.88 (27.34)	35.68 (10.68)	63.86 (12.57)
Total chlorophyll (µg g^−1^ FW)	491.9 (112.0)	182.3 (28.8)	447.4 (22.8)	885.1 (174.7)	597.4 (60.7)	852.4 (158.6)
Total carotenoids (µg g^−1^ FW)	125.4 (30.8)	57.6 (6.8)	107.1 (16.9)	132.0 (46.2)	159.1 (5.4)	142.2 (33.2)
Fruit weight (g)	1.92 (0.07)	2.12 (0.32)	2.14 (0.17)	2.66 (0.40)	2.12 (0.08)	2.33 (0.32)
Stone-to-pulp ratio (%)	30.00 (1.14)	29.51 (5.41)	34.54 (1.45)	26.93 (5.84)	25.83 (1.81)	25.30 (0.80)
Pulp water content (%)	52.79 (1.62)	55.14 (9.97)	46.06 (1.98)	58.06 (13.75)	62.93 (2.50)	64.78 (1.64)
Fruit-size index (cm^2^)	1.054 (0.335)	0.914 (0.323)	1.834 (1.174)	0.959 (0.464)	0.977 (0.215)	0.716 (0.251)

Beth = Bethlehem; Heb = Hebron; Ram = Ramallah; I, II and III denote the broad 50–100, 25–50 and 15–20-year operational age ranges, respectively. Exact within-class ages were not available. Pigment concentrations were recalculated from the original absorbance readings (Section 2.5.2); carotenoid means are based on *n* = 7 for Bethlehem Range I and *n* = 13 for Ramallah Range II after exclusion of one physically impossible estimate in each group.

**Table 4:** Photosynthetic-pigment concentrations (mean ± SD) and carotenoid-to-chlorophyll ratios by governorate × age-range group.

Group	Total Chlorophyll (µg g^−1^ FW)	Total Carotenoids (µg g^−1^ FW)	Carotenoid:Chlorophyll Ratio (Extract-Level)
Bethlehem_RangeI	491.9 ± 112.0	125.4 ± 30.8	0.246 ± 0.019
Bethlehem_RangeII	182.3 ± 28.8	57.6 ± 6.8	0.318 ± 0.023
Hebron_RangeI	447.4 ± 22.8	107.1 ± 16.9	0.240 ± 0.041
Hebron_RangeII	885.1 ± 174.7	132.0 ± 46.2	0.162 ± 0.081
Hebron_RangeIII	597.4 ± 60.7	159.1 ± 5.4	0.268 ± 0.018
Ramallah_RangeII	852.4 ± 158.6	142.2 ± 33.2	0.180 ± 0.063

Values are mean ± SD of extract-level concentrations recalculated from the original absorbance readings with Equations (3)–(6) (Section 2.5.2); *n* = 8, 3, 3, 5, 5 and 14 extracts in table order for total chlorophyll, and *n* = 7 (Bethlehem Range I) and *n* = 13 (Ramallah Range II) for carotenoids and the ratio after exclusion of one physically impossible estimate in each group. Values are descriptive; no omnibus or pairwise tests are reported because extracts were nested within orchards and unevenly distributed among groups (Section 2.7). Chlorophyll *a* and *b* fractions are reported in Table S3.

**Table 5:** Descriptive synthesis of the Range II governorate comparison and exploratory relationships with symptomatic leaf-spot incidence.

Trait	Range II Governorate Comparison	Relationship with Symptomatic Incidence	Interpretation
Suspected Cercospora leaf-spot incidence	Similar means (9.95–11.02%); distributions overlapped	—	Highest mean in Bethlehem Range I; the unbalanced, broad age-class structure prevents attribution to age or governorate.
Leaf water content	Higher mean in Bethlehem (87.5%) than in Hebron and Ramallah (67.0–71.1%)	No clear association (orchard level)	Leaf hydration differed among sampled governorates within Range II
Photosynthetic pigments	Lower mean total chlorophyll in Bethlehem (182 µg g^−1^ FW) than in Hebron and Ramallah (852–885 µg g^−1^ FW)	No clear association (group level)	Total chlorophyll differed among governorates within Range II
Total phenolic content	Overlapping means (47.6–63.9 mg GAE g^−1^ DW) with large SDs	No clear association (orchard level)	No clear association with symptomatic incidence; group differences cannot be attributed to age or governorate
Fruit characteristics (weight, size)	Fruit weight highest in Hebron (2.66 g); fruit-size index lowest in Ramallah (0.716 cm^2^), with overlapping distributions	No clear association (orchard level)	Fruit weight differed among governorates within the broad Range II class; climatic, soil, management and sampling influences cannot be separated.
Stone-to-pulp ratio	Means 29.51% (Bethlehem), 26.93% (Hebron) and 25.30% (Ramallah)	Negative trend; non-significant	Exploratory tendency only; no statistically supported association with symptomatic incidence.
Pulp water content	Means 55.14% (Bethlehem), 58.06% (Hebron) and 64.78% (Ramallah)	Positive trend; non-significant	Largest positive descriptive coefficient with symptomatic incidence, but not statistically significant.

## Data Availability

The data supporting the findings of this study are available from the corresponding author upon reasonable request.

## Supplementary Materials

The following supporting information can be downloaded at: https://www.mdpi.com/article/10.3390/jof12100792/s1, Table S1, descriptive statistics by governorate × age-range group; Table S2, pairwise differences between group means; Table S3, individual chlorophyll fractions; Table S4, descriptive summary of the Range II comparison; Table S5, exploratory correlation summary.
