# Supplementary material for: Variation in Suspected Cercospora Leaf Spot Incidence and Host Physiological and Fruit Traits Across Olive Tree Age Classes and Governorates in Palestinian Orchards
Source: J Fungi (Basel). 2026 Oct 21;12(10):792. doi: 10.3390/jof12100792 (PMC13705752; doi:10.3390/jof12100792)
Supplement: Supplementary file 1 [file jof-12-00792-s001.zip › jof-4534731-supplementary.pdf]

Supplementary Materials

Variation in Suspected Cercospora Leaf Spot Incidence and Host Physiological and Fruit Traits Across Olive Tree Age Classes and Governorates in Palestinian Orchards

Ruba Al-Nazer [1] and Besma Sghaier-Hammami [1,*]

Table S1. Descriptive statistics for the nine analytical domains in each governorate × age-range group: number of measurement records (n), mean, standard deviation (SD) and coefficient of variation (CV).

Statistic	Bethlehem I	Bethlehem II	Hebron I	Hebron II	Hebron III	Ramallah II
**Symptomatic leaf-spot incidence (%); branch records**						
n	46	74	77	76	40	116
Mean	14.11	9.95	9.29	10.89	9.93	11.02
SD	5.73	5.07	4.11	4.67	4.39	5.70
CV (%)	40.6	51.0	44.2	42.9	44.2	51.7
**Leaf relative water content (%); leaf samples**						
n	5	10	5	18	5	15
Mean	82.85	87.52	64.95	71.07	68.32	67.00
SD	7.15	5.87	6.27	7.16	12.00	8.20
CV (%)	8.6	6.7	9.7	10.1	17.6	12.2
**Total phenolic content (mg GAE g^{-1} DW); leaf samples**						
n	4	9	5	8	10	12
Mean	61.75	47.61	52.94	51.88	35.68	63.86
SD	15.25	13.50	26.70	27.34	10.68	12.57
CV (%)	24.7	28.4	50.4	52.7	29.9	19.7
**Total chlorophyll (µg g^{-1} FW); extracts**						
n	8	3	3	5	5	14
Mean	491.9	182.3	447.4	885.1	597.4	852.4
SD	112.0	28.8	22.8	174.7	60.7	158.6
CV (%)	22.8	15.8	5.1	19.7	10.2	18.6
**Total carotenoids (µg g^{-1} FW); extracts**						
n	7	3	3	5	5	13
Mean	125.4	57.6	107.1	132.0	159.1	142.2
SD	30.8	6.8	16.9	46.2	5.4	33.2
CV (%)	24.6	11.8	15.8	35.0	3.4	23.3
**Fruit weight (g); ten-fruit composites**						
n	5	10	5	16	5	15
Mean	1.92	2.12	2.14	2.66	2.12	2.33
SD	0.07	0.32	0.17	0.40	0.08	0.32
CV (%)	3.6	15.1	7.9	15.0	3.8	13.7
**Stone-to-pulp ratio (%); ten-fruit composites**						
n	5	10	5	16	5	15
Mean	30.00	29.51	34.54	26.93	25.83	25.30
SD	1.14	5.41	1.45	5.84	1.81	0.80
CV (%)	3.8	18.3	4.2	21.7	7.0	3.2
**Pulp water content (%); ten-fruit composites**						
n	5	10	5	16	5	15
Mean	52.79	55.14	46.06	58.06	62.93	64.78
SD	1.62	9.97	1.98	13.75	2.50	1.64

Statistic	Bethlehem I	Bethlehem II	Hebron I	Hebron II	Hebron III	Ramallah II
CV (%)	3.1	18.1	4.3	23.7	4.0	2.5
**Fruit-size index (cm^2); individual fruits**						
n	250	449	500	740	300	750
Mean	1.054	0.914	1.834	0.959	0.977	0.716
SD	0.335	0.323	1.174	0.464	0.215	0.251
CV (%)	31.8	35.3	64.0	48.4	22.0	35.1

All records were obtained from the 45 tagged trees (five per orchard) in the nine orchards. n is the number of measurement records (branches, leaf samples, extracts, ten-fruit composites or individual fruits); because these records are nested within trees and orchards, n is not the number of independent biological replicates and no inferential statistics are reported. CV = 100 × SD / mean. Carotenoid values exclude one physically impossible estimate in Bethlehem Range I and one in Ramallah Range II.

Table S2. Pairwise differences between group means (group I − group J) for seven analytical domains, presented descriptively.

(I) Group	(J) Group	Incidence (%)	RWC (%)	Total Chl (µg g^{-1} FW)	TPC (mg GAE g^{-1} DW)	FSI (cm^2)	FW (g)	PWC (%)
Bethlehem_RangeI	Bethlehem_RangeII	4.166	−4.665	309.505	14.139	0.141	−0.205	−2.345
Bethlehem_RangeI	Hebron_RangeI	4.828	17.902	44.481	8.808	−0.779	−0.216	6.727
Bethlehem_RangeI	Hebron_RangeII	3.228	11.778	−393.201	9.867	0.096	−0.736	−5.267
Bethlehem_RangeI	Hebron_RangeIII	4.187	14.534	−105.562	26.069	0.077	−0.198	−10.138
Bethlehem_RangeI	Ramallah_RangeII	3.089	15.853	−360.589	−2.115	0.338	−0.411	−11.990
Bethlehem_RangeII	Hebron_RangeI	0.662	22.567	−265.024	−5.331	−0.920	−0.011	9.072
Bethlehem_RangeII	Hebron_RangeII	−0.938	16.443	−702.706	−4.272	−0.045	−0.531	−2.921
Bethlehem_RangeII	Hebron_RangeIII	0.022	19.199	−415.067	11.930	−0.064	0.007	−7.793
Bethlehem_RangeII	Ramallah_RangeII	−1.076	20.519	−670.094	−16.253	0.198	−0.206	−9.644
Hebron_RangeI	Hebron_RangeII	−1.600	−6.124	−437.682	1.059	0.875	−0.519	−11.994
Hebron_RangeI	Hebron_RangeIII	−0.640	−3.368	−150.043	17.260	0.857	0.018	−16.865
Hebron_RangeI	Ramallah_RangeII	−1.738	−2.049	−405.070	−10.923	1.118	−0.195	−18.717
Hebron_RangeII	Hebron_RangeIII	0.959	2.756	287.639	16.202	−0.019	0.537	−4.871
Hebron_RangeII	Ramallah_RangeII	−0.139	4.075	32.612	−11.982	0.243	0.324	−6.723
Hebron_RangeIII	Ramallah_RangeII	−1.098	1.319	−255.027	−28.183	0.261	−0.213	−1.852

RWC = leaf relative water content; Chl = chlorophyll; TPC = total phenolic content; FSI = fruit-size index; FW = fruit weight; PWC = pulp water content. Differences were calculated from the unrounded group means. They are descriptive only: no standard errors, pairwise tests or significance levels are reported because branches, fruits, extracts and composite samples are subsamples nested within trees and orchards. Differences for total carotenoids and stone-to-pulp ratio can be obtained from the means in Table S1.

Table S3. Individual chlorophyll fractions (mean ± SD, µg g^{-1} FW) and chlorophyll *a*/*b* ratios by governorate × age-range group.

Group	Chlorophyll *a*	Chlorophyll *b*	Chl *a*/*b* ratio
**Bethlehem_RangeI**	445.3 ± 85.6	91.5 ± 25.6	4.97 ± 0.66
**Bethlehem_RangeII**	164.9 ± 28.9	28.3 ± 2.9	5.84 ± 0.80
**Hebron_RangeI**	382.6 ± 30.0	90.1 ± 20.9	4.47 ± 1.46
**Hebron_RangeII**	606.2 ± 14.5	321.1 ± 170.6	2.49 ± 1.59
**Hebron_RangeIII**	508.5 ± 35.1	122.7 ± 29.4	4.27 ± 0.66
**Ramallah_RangeII**	593.7 ± 43.9	300.0 ± 140.4	2.38 ± 1.01

Values are extract-level estimates calculated with Equations (3) and (4) of the main text (Section 2.5.2) from the absorbances at 663 and 646 nm and expressed on the same fresh-weight basis as total chlorophyll (n = 8, 3, 3, 5, 5 and 14 extracts in table

order; n = 7 for chlorophyll *b* and the *a*/*b* ratio in Bethlehem Range I after exclusion of one negative chlorophyll *b* estimate). Total chlorophyll in the main text (Table 4) was calculated directly with Equation (6) and not as the sum of the two fractions; the sum of chlorophyll *a* and *b* is therefore approximately 5–9% higher than the reported total chlorophyll. The fractions are reported for descriptive completeness only.

Table S4. Descriptive summary of the Range II comparison: group means with standard deviations in parentheses and numbers of records (n) for the three governorates within the shared 25–50-year age class.

Domain	Bethlehem II	Hebron II	Ramallah II	Highest − lowest mean
**Symptomatic leaf-spot incidence (%)**	9.95 (5.07); n = 74	10.89 (4.67); n = 76	11.02 (5.70); n = 116	1.08
**Leaf relative water content (%)**	87.52 (5.87); n = 10	71.07 (7.16); n = 18	67.00 (8.20); n = 15	20.52
**Total phenolic content (mg GAE g^{-1} DW)**	47.61 (13.50); n = 9	51.88 (27.34); n = 8	63.86 (12.57); n = 12	16.25
**Total chlorophyll (µg g^{-1} FW)**	182.3 (28.8); n = 3	885.1 (174.7); n = 5	852.4 (158.6); n = 14	702.7
**Total carotenoids (µg g^{-1} FW)**	57.6 (6.8); n = 3	132.0 (46.2); n = 5	142.2 (33.2); n = 13	84.6
**Fruit weight (g)**	2.12 (0.32); n = 10	2.66 (0.40); n = 16	2.33 (0.32); n = 15	0.53
**Stone-to-pulp ratio (%)**	29.51 (5.41); n = 10	26.93 (5.84); n = 16	25.30 (0.80); n = 15	4.21
**Pulp water content (%)**	55.14 (9.97); n = 10	58.06 (13.75); n = 16	64.78 (1.64); n = 15	9.64
**Fruit-size index (cm^2)**	0.914 (0.323); n = 449	0.959 (0.464); n = 740	0.716 (0.251); n = 750	0.243

The last column gives the difference between the highest and the lowest of the three group means. The comparison is restricted to one broad age class and is descriptive; it is neither exact age standardization nor statistical control of tree age, and no tests are reported because the records are nested within trees and orchards.

Table S5. Exploratory Spearman rank-order correlations of total chlorophyll and of symptomatic leaf-spot incidence with the other domains, calculated from the six governorate × age-range group means (group level) and from orchard means (orchard level).

Domain	ρ vs total chlorophyll (group level, n = 6)	*P*	ρ vs incidence (group level, n = 6)	*P*	ρ vs incidence (orchard level)	*P*	n (orchards)
**Symptomatic leaf-spot incidence**	+0.371	0.468	—	—	—	—	—
**Leaf relative water content**	−0.257	0.623	+0.429	0.397	−0.100	0.798	9
**Total phenolic content**	+0.200	0.704	+0.600	0.208	+0.383	0.308	9
**Total chlorophyll**	—	—	+0.371	0.468	n.d.	—	—
**Total carotenoids**	+0.771	0.072	+0.143	0.787	n.d.	—	—
**Fruit weight**	+0.543	0.266	−0.086	0.872	+0.214	0.645	7
**Stone-to-pulp ratio**	−0.657	0.156	−0.257	0.623	−0.536	0.215	7
**Pulp water content**	+0.657	0.156	+0.257	0.623	+0.607	0.148	7
**Fruit-size index**	−0.314	0.544	−0.371	0.468	−0.183	0.637	9

n.d. = not determined at orchard level; n (orchards) = number of orchards with matched data for the orchard-level coefficient. With n = 6–9 aggregated values, the coefficients and their nominal *P* values are descriptive indications only and none is statistically significant at $P = 0.05$. Group-level and orchard-level coefficients are based on different aggregation units and need not agree. Correlations involving fruit traits are cross-seasonal, linking the October–November 2024 harvest with the spring 2025 leaf campaign.
